# Beyond Single Diagnosis: Exploring Multidiagnostic Realities in Pediatric Patients through Genome Sequencing

**DOI:** 10.1155/2024/9115364

**Published:** 2024-04-23

**Authors:** Fen Guo, Ruby Liu, Yinghong Pan, Mary Colasanto, Christin Collins, Madhuri Hegde

**Affiliations:** ^1^Revvity Omics, Pittsburgh, Pennsylvania, USA; ^2^Department of Obstetrics, Gynecology and Reproductive Sciences, University of Pittsburgh, Pittsburgh, Pennsylvania, USA

## Abstract

Recent advancements in the next-generation sequencing have illuminated the occurrence of multiple genetic diagnoses (MGD). While exome sequencing has provided insights, genome sequencing (GS), the most comprehensive diagnostic tool, remains underexplored for studying MGD prevalence. We retrospectively analyzed 1487 pediatric cases from our laboratory, employing GS to investigate the incidence of single definitive genetic diagnosis (SDD) and MGD in children suspected of having a genetic disease. Of these patients, 273 received at least one definitive diagnosis, including 245 with SDD (16.5%) and 28 with MGD (1.9%). Diagnostic yield was consistent across genders and unaffected by previous testing in SDD cases. Notably, prior testing significantly increased the diagnostic yield in MGD cases to 2.7% overall and 14.4% among diagnosed cases, compared to 1.1% for those with GS as a first-tier test. Age was a significant factor in diagnostic outcome for both SDD and MGD cases with neonates showing the highest diagnostic yield of 24.5% in SDD and a notably higher yield in MGD at 4.9%, representing 16.7% of the diagnosed cases. Of the 28 MGD cases, 17 exhibited distinct phenotypes, 9 had overlapping features, and 2 presented a mix, underscoring the genetic and phenotypic heterogeneity within this group. This study is the first to exclusively use GS to assess MGD prevalence. Our findings highlight the complexity of rare diseases and emphasize the importance of comprehensive, genome-level diagnostics. Clinicians must ensure that diagnoses fully account for the observed phenotypes to inform optimal therapeutic strategies and management.

## 1. Introduction

Despite their diverse characteristics and geographically dispersed occurrence, rare diseases collectively exhibit a global prevalence of 3.5%-5.9%. Of these conditions, 70% have a genetic basis and another 70% are associated with pediatric onset [1]. These conditions predominantly manifest as a single genetic disorder, yet in certain cases, clinical features extend beyond the classic symptomatology attributed to the established genetic cause. This discrepancy may signify either an expanded phenotype of the original condition or a distinct, coexisting diagnosis [2]. The discernment between phenotype expansion and comorbidity grows increasingly complex when multiple diagnoses converge into a nuanced clinical presentation [3].

Over the last decade, a substantial influx of data has elucidated the incidence of multiple genetic diagnoses (MGD), largely propelled by advances in the next-generation sequencing (NGS) technologies. NGS permits simultaneous scrutiny of an extensive array of genes, the complete exome, or even the entire genome. According to data derived from exome sequencing (ES), the prevalence of MGD varies between 3.5-7.2% in diagnostic scenarios and 0.4-4.0% in overall cases, with fluctuations based on cohort size and population demographics [4]. Given the technical superiority of genome sequencing (GS) over ES in delivering enhanced diagnostic yield [5], it is plausible that rates of MGD could surpass previous estimates.

In this study, we undertook an exhaustive retrospective analysis of 1487 pediatric cases subjected to clinical diagnostic GS [6]. We aimed to determine the incidence of both isolated and multiple diagnoses within a pediatric cohort with clinical suspicion of a genetic disorder. To our knowledge, this study is the first to assess the prevalence of MGD exclusively using GS data, potentially offering a more accurate snapshot of the true incidence in the clinical setting.

## 2. Materials and Methods

### 2.1. Study Design and Participants

A retrospective analysis was performed on consecutive pediatric clinical GS cases referred to our laboratory between March 2018 and September 2022 without imposing specific inclusion or exclusion criteria. A parent or legal guardian of all patients included in this analysis provided written informed consent for research use of deidentified data. All procedures were executed within the framework of Revvity Omics.

### 2.2. Sequencing Data Generation and Analysis

GS was performed using a PCR-free methodology, as described previously [7, 8]. Briefly, sequence libraries were prepared from isolated genomic DNA using the NEXTFLEX Rapid XP kit (PerkinElmer) and sequenced on the NovaSeq 6000 (Illumina Inc.). Raw data was demultiplexed and converted to FASTQ format using the Illumina bcl2fastq2 converter (Illumina Inc.). Sequences were aligned to the human reference genome GRCh37 (hg19), and variant calling was completed using the Illumina Bio-IT DRAGEN Platform (Illumina Inc.). The average coverage of the nuclear and mitochondrial genomes was 40x and 1000x, respectively. Repeat expansion analysis for 31 disorders (Supplementary Table 1) was carried out using ExpansionHunter (default settings, Illumina Inc.) [8, 9]. Screening for spinal muscular atrophy (SMA) was performed using in-house bioinformatic tools adapted from published literature with modifications [8, 10].

Single nucleotide variants (SNVs) and small insertions and deletions (indels) were annotated by Revvity Omics' proprietary Ordered Data Interpretation Network (ODIN), which integrates multiple databases, including HGMD, gnomAD, and ClinVar, and tags curated intronic pathogenic variants for analysis. Variant classification was performed based on American College of Medical Genetics and Genomics (ACMG) guidelines [11, 12] with additional triage of intronic and intergenic regions according to clinical information and inheritance patterns. Copy number variant (CNV) analysis was conducted using NxClinical (Bionano), which applies a read-depth strategy, and by utilizing genome maps from DGV, DECIPHER, and ClinGen for assessment [13].

### 2.3. Clinical GS Testing and Reporting Policy

GS serves as a diagnostic tool for individuals with suspected rare genetic disorders, offering proband-only to trio-based tests with parents. The testing encompasses sequencing of the nuclear and mitochondrial genomes, screening for repeat expansion disorders, and SMA analysis. Variants classified as pathogenic (P), likely pathogenic (LP), or uncertain significance (VUS) concomitant with the clinical phenotype, as provided by the ordering provider, were reported. Screening results for repeat expansion disorders were evaluated and reported if pertinent to the clinical phenotype, following a “rule-out” strategy [8, 14]. SMA analysis was conducted when relevant to the clinical phenotype or when consent for carrier status reporting was obtained. Both screening approaches have been standard for all cases sequenced after November 2021 and were not applied retroactively. In addition to primary diagnostic findings, patients can consent to receive secondary findings, including ACMG-recommended genes [15–18], pharmacogenomic variants (PGx), carrier status, and other diagnostic findings. Reported PGx variants include selected allele haplotypes that have been recommended by the Association for Molecular Pathology pharmacogenomics working group and are classified as Clinical Pharmacogenetics Implementation Consortium (CPIC) level A and PharmGKB level 1A (Supplementary Table 2).

Carrier status is reported for 341 genes associated with autosomal recessive (AR) and X-linked (XL) gene disorders (Supplementary Table 3). Other diagnostic findings include all pathogenic (P or LP) findings in genes associated with diseases that are unrelated to indications for testing. Most diseases in this category manifest as reduced penetrance or late onset.

### 2.4. Diagnostic Case Category Definition

A definitive diagnosis (DD) refers to any case with a pathogenic finding aligned with the patient's clinical phenotype. Such cases are further delineated into three categories for analytical clarification: (1) single definitive diagnosis (SDD) that describes cases where only a single diagnostic finding was identified; (2) definitive multiple genetic diagnosis (DMGD) that denotes cases with two or more pathogenic findings associated with distinct genetic diseases concordant with the clinical phenotype; and (3) presumed multiple genetic diagnosis (PMGD) that describes cases with a pathogenic finding and a VUS associated with distinct genetic diseases that aligned with the clinical presentation. Cases categorized as DMGD and PMGD were collectively evaluated as MGD.

### 2.5. Statistical Analysis

Statistical analysis was performed to assess the distribution and significance of various demographic and clinical variables. Fisher's exact test was performed to evaluate the significance of differences in diagnostic yield between sex and history of previous testing. The chi-square test was performed to evaluate the significance of differences in diagnostic yield across age groups. The statistical significance was set at *P* < 0.05, two-tailed. Tests were executed using GraphPad Prism 10.1.0, and results were reported with 95% confidence intervals where applicable.

## 3. Results

### 3.1. Cohort Characteristics

The cohort of 1487 pediatric patients included 647 females (43.5%) and 840 males (56.5%). The age distribution ranged from neonates to adolescents, with the largest group being children aged 3-11 years (44.2%, 657/1487). The case categories were almost evenly split between nontrio (47.8%, 711/1487) and trio (52.2%, 776/1487) setups. Prior genetic testing was conducted for 694 patients (46.7%), while 793 patients (53.3%) had GS as a first-tier test. The distribution of demographic information across various age groups is detailed in Table 1.

### 3.2. Overall Diagnostic Rate for SDD and MGD

**Figure 1:**
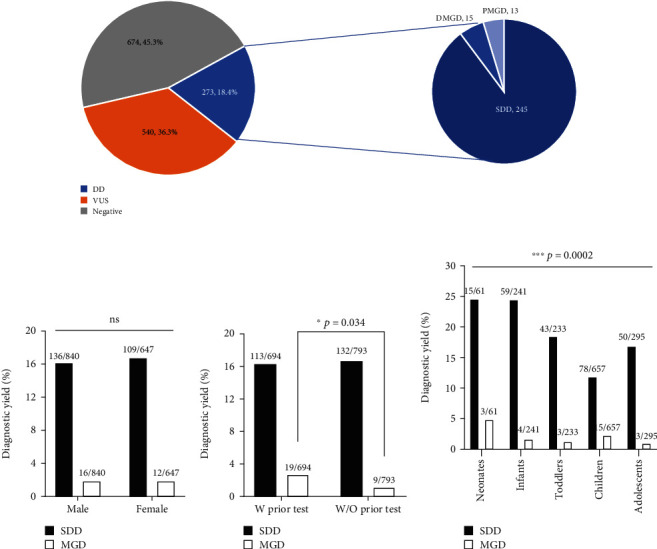
Overall diagnostic rate and variables affecting diagnostic yield in SDD and MGD cases: (a) overall diagnostic rate for SDD and MGD; (b) diagnostic yield vs. sex; (c) diagnostic yield vs. with (W) or without (W/O) prior testing; (d) diagnostic yield vs. age group. Abbreviations: DD: definitive diagnosis; VUS: variants of uncertain significance; SDD: single definitive diagnosis; MGD: multiple genetic diagnoses; DMGD: definitive multiple genetic diagnoses; PMGD: presumed multiple genetic diagnoses; ns: not significant; ^∗^*p* value < 0.05; ^∗∗∗^*p* value < 0.001.

A definitive diagnosis was made in 273 of 1487 patients, corresponding to a diagnostic yield of 18.4%. Of these, 245 were SDD cases, comprising 16.5% (245/1487) of the total cohort and 89.7% (245/273) of the diagnosed cases. The remaining 28 cases were MGD, totaling 1.9% (28/1487) of the cohort and 10.3% (28/273) of the diagnostic cases (Figure 1(a)). The MGD cases included 15 DMGD cases [1.0% (15/1487) overall, 5.5% (15/273) among diagnosed; Table 2 and Supplementary Table 4] and 13 PMGD cases [0.9% (13/1487) overall, 4.8% (13/273) among diagnosed; Table 3 and Supplementary Table 5]. Additionally, 9 cases were identified with at least one definitive diagnostic finding and one secondary finding (Supplementary Table 6).

### 3.3. Variables Affecting Diagnostic Yield in SDD and MGD Cases

Diagnostic yield showed no difference between male and female patients in SDD and MGD cases. In SDD cases, the yield was 16.2% (136/840) for males and 16.8% (109/647) for females. For MGD, it was 1.9% for both (Figure 1(b)). Similarly, the diagnostic yield for SDD cases was unrelated to the previous testing: 16.3% (113/694) for those with prior testing and 16.6% (132/793) for those without. Remarkably, a statistically significant disparity emerged in the diagnostic yield among MGD cases: individuals with prior genetic testing showed notably higher diagnostic yield with 2.7% (19/694) overall and 14.4% (19/130) among diagnosed compared to those with GS as a first-tier genetic test (1.1% (9/793) overall and 6.4% (9/143) among diagnosed) (Figure 1(c)). Importantly, 17 of the 19 MGD cases with prior genetic testing failed to receive a complete diagnosis: 8 patients received a partial diagnosis, and testing was nondiagnostic in 9 patients. Subsequent GS was necessary to delineate the remaining, unexplained phenotypes. In 2 cases, GS confirmed prior results, suggesting that further studies like transcriptomic or epigenetic analyses may be needed.

Age at testing also imposed a significant impact on diagnostic yield (Figure 1(d)). Specifically, neonates and infants exhibited the highest diagnostic yields for SDD at 24.6% (15/61) and 24.5% (59/241), respectively. A similar trend was observed for MGD cases with neonates showing a markedly elevated diagnostic yield of 4.9% (3/61), accounting for 16.7% (3/18) of the diagnosed cases. This significantly exceeded the diagnostic yield in all other age groups, which fluctuated between 1.3% and 2.3%.

### 3.4. Complex Landscape of Definitive Diagnosed Cases: Variant Types, Inheritance Patterns, and Phenotypic Diversity

**Figure 2:**
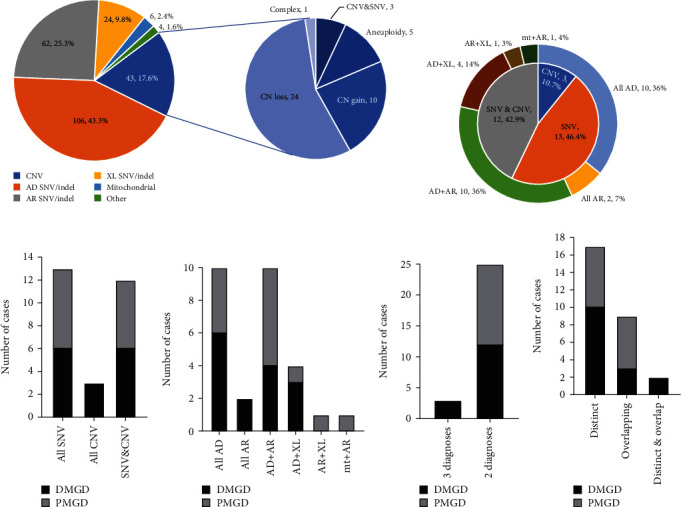
The complex landscape of definitive diagnosed cases by variant types of (a) SDD and (b) MGD. Detailed subcategory comparisons in MGD cases: (c) variant types, (d) inheritance patterns, (e) diagnosis numbers, and (f) phenotypic complexity. Abbreviations: SDD: single definitive diagnosis; MGD: multiple genetic diagnoses; SNV: single nucleotide variants; CNV: copy number variants; AD: autosomal dominant; AR: autosomal recessive; XL: X-linked; DMGD: definitive multiple genetic diagnoses; PMGD: presumed multiple genetic diagnoses.

In SDD cases, 79% of diagnoses were attributed to SNVs or indels. These variants predominantly manifested in autosomal dominant (AD, 43.3%, 106/245) and AR (25.3%, 62/245) genes, while XL genes contributed to 9.8% (24/245) of the cases. CNVs accounted for 16.3% (40/245) of the diagnostic variants and included aneuploidies (5), gains (10), losses (24), and a complex rearrangement. Additionally, three cases were compound heterozygous for an SNV and CNV in an AR gene. SMA and repeat expansion disorder screening each identified 2 positive cases. Mitochondrial variants were identified in 2.4% (6/245) of cases (Figure 2(a)).

In contrast, the MGD cases were comprised of a more complex genetic landscape. Genetic diagnoses in 46.4% (13/28) of cases were exclusively due to SNVs while 42.9% (12/28) of cases received genetic diagnoses based on a combination of both SNVs and CNVs for each diagnosis. In addition, a minority (3/28, 10.7%) of patients had diagnoses purely based on CNVs (Figures 2(b) and 2(c)). A single mode of inheritance was noted in 42.9% (12/28) of cases, and 57.1% (16/28) of patients showed multiple inheritance types (Figures 2(b) and 2(d)). Most patients had one additional diagnosis (25/28, 89.3%), and a minority had three distinct diagnoses (3/28, 10.7%) (Figure 2(e)). Furthermore, the diversity within this cohort eloquently demonstrates the phenotypic complexity of MGD cases: 17 presented with distinct features of each of their diagnoses, 9 showed traits that overlapped between their diagnoses, and 2 displayed a mix of distinct and overlapping features (Figure 2(f)). This finding underscores the necessity for a comprehensive diagnostic approach to capture the multitude of variation underlying complex genetic conditions.

### 3.5. Illustrative Case Presentation

*Case PKIG00063* was an 11-year-old male with a known diagnosis of trisomy 21 and a marker chromosome originating from 2q11.1 to 2q12.1, initially identified by karyotype and chromosomal microarray. In addition to the classic features of Down syndrome, this individual presented with other unexplained clinical features including intrauterine growth restriction (IUGR), cerebral atrophy, cerebellar and brain stem volume loss, cataract, microphthalmia, hemiparesis, and nephrotic syndrome, including hypertension, proteinuria, electrolyte abnormalities, and renal failure. The unexplained severe and complicated clinical features indicated the possibility of an additional underlying etiology. GS was pursued, and the data not only confirmed the initial diagnosis of trisomy 21 and copy number gain of 2q11.1q12.1 but also identified uniparental isodisomy (UPD) of chromosome 5 and a homozygous likely pathogenic variant in *ERCC8* [NM_000082.3:c.613G>C p.(Ala205Pro)]. Homozygous or compound heterozygous pathogenic variants in the *ERCC8* gene have been associated with Cockayne syndrome type A (OMIM 216400). Since the *ERCC8* gene is located on chromosome 5, we thereby confirmed a diagnosis of an AR condition due to UPD, which explained the severe neurodegeneration and abnormal ocular and renal manifestations presented in this patient. The identification of UPD5 also provides valuable information for understanding the parental reproductive risk regarding AR Cockayne syndrome.

*Case PKIG00155* was a 3-year-old female with a history of complex febrile seizures, rhizomelic shortening of the proximal long bones, narrow thorax, lumbar kyphosis, brachydactyly, and macrocephaly. Clinical suspicion included hypochondroplasia/achondroplasia caused by *FGFR3*-related skeletal dysplasia. Previous karyotype testing and *FGFR3* gene sequencing were unremarkable. GS identified a pathogenic variant in *COL2A1* [NM_001844.4:c.2257G>A p.(Gly753Ser)] and a pathogenic variant in *PCDH19* [NM_001184880.1:c.2571_2572delGC p.?]. Pathogenic *COL2A1* variants have been associated with varying skeletal dysplasias, including achondrogenesis and hypochondrogenesis (OMIM 200610) [19], while pathogenic *PCDH19* variants have been associated with a female-restricted form of sporadic infantile epileptic encephalopathy (OMIM 300088) [20]. The consolidated findings delineated two distinct genetic etiologies that contributed to this patient's full clinical phenotype.

*Case PKIG02101* was a 7-day-old male that presented on the first day of life with severe hyperammonemia, lactic acidosis, seizures, arrhythmia, respiratory failure, apnea, neonatal jaundice, hyperkalemia, hypomagnesemia, foot deformity, anuria, oliguria, abnormal kidney function, abnormal phosphorus metabolism, abnormal liver function, and hypotension. GS was performed, and two findings associated with submitted clinical manifestations were identified: a pathogenic 600.5 kb contiguous deletion of Xp11.4, including the *SRPX*, *RPGR*, *OTC*, *TSPAN7*, and *MID1IP1* genes, associated with severe ornithine transcarbamylase (OTC) deficiency in males [21, 22] and a likely pathogenic variant in *RORB* [NM_006914.4:c.286G>T p.(Glu96Ter)], associated with pediatric-onset idiopathic generalized and absence epilepsy. Although routine GS with a turnaround time of about 4 weeks was requested, the results were delivered to the ordering provider in 7 days. These timely results provided a survival opportunity for this critically ill infant, potentially averting severe complications through interventions like liver transplant [23]. Cascade parental studies will support recurrence risk assessment and provide appropriate medical and prophylactic management.

*Case PKIG01849* was a 2-month-old male evaluated for hypertonia of the upper extremities; growth restriction, including IUGR and small for gestational age; failure to thrive; and microcephaly with prenatal onset. He had low-set, posteriorly rotated ears and abdominal issues, including poor feeding and gastroesophageal reflux. Other concerns included underdevelopment of the inferior vermis, cryptorchidism, and aortic root dilation. Family history revealed both parents were obligate carriers of phenylketonuria (PKU). Prenatal genetic testing disclosed that the baby carried the *PAH* [NM_000277.1:c.527G>T p.(Arg176Leu)] and [NM_000277.1:c.898G>T p.(Ala300Ser)] pathogenic variants in *trans.* However, PKU does not explain all of the clinical features present in this patient. Trio GS identified a *de novo KAT6A* [NM_006766.3:c.3385C>T p.(Arg1129Ter)] pathogenic variant in addition to the *PAH* findings. The *KAT6A* gene, located on chromosome 11, encodes a lysine acetyltransferase that regulates gene transcription and expression. Patients with *KAT6A* pathogenic variants have Arboleda-Tham syndrome (OMIM 616268), an AD disease characterized by microcephaly, hypertonia, cardiac anomalies, gastrointestinal reflux, feeding difficulties, failure to thrive, and developmental delay [24, 25]. The nature of the *KAT6A* variant and the clinical presentation of this patient align well with Arboleda-Tham syndrome. The new molecular finding provides an additional, distinct clinical diagnosis and guides medical management and prophylactic strategies, allowing this family to start appropriate treatment in the early stages, which may result in better outcomes.

*Case PKIG01041* was a 4-year-old female referred by a genetic clinic for neurological complaints, including partially empty sella, developmental delay, right-sided weakness, and hypotonia. Family history was noncontributory. Trio GS was performed, and two findings were identified. The first was a *de novo* likely pathogenic variant in *CUL3* (NM_003590.4:c.264+1G>C p.?). *CUL3* is associated with a neurodevelopmental disorder (OMIM 619239) characterized by autism spectrum disorder, developmental delay, and cognitive developmental impairment with variable severity and age of onset [26–28]. In addition, patients with *CUL3* pathogenic variants may have seizures, congenital heart defects, dysmorphic facial features, and abnormal brain imaging [29]. The second finding in this patient was a *de novo* 22q11.2 duplication. Chromosome 22q11.2 duplication syndrome (OMIM 608363) is a well-documented pathogenic finding with variable expressivity and incomplete penetrance. The clinical features include psychomotor developmental delay, heart defects, velopharyngeal insufficiency, and muscular hypotonia. Both findings in this patient explained the delayed neurodevelopment and provided molecular and medical evidence for her individualized education program (IEP) and the severity of the symptoms. In addition, the 22q11.2 microduplication syndrome diagnosis supports access to a comprehensive cardiac evaluation, which according to the submitted clinical information had not been conducted before GS.

## 4. Discussion

The inherent difficulty in diagnosing rare genetic disorders is often underestimated due to the synergy of numerous genetic factors and diverse clinical symptoms complicating the diagnostic process [30–34]. To tackle this challenge, an exhaustive and methodological approach is required to uncover their multifaceted genetic basis and consequential clinical implications. We conducted an extensive retrospective analysis of 1487 pediatric cases, making this the first study to explore the prevalence of MGD using GS, the most advanced diagnostic assay currently available.

With a definitive diagnostic yield of 18.4%, we observed that MGD cases constituted 10.3% of the diagnostic cohort and 1.9% of all cases. Although the incidence of MGD in our cohort is higher than previous ES studies on dual diagnoses, this finding may still be under-representative given GS's superior diagnostic yield. Comparing diagnostic rates between laboratories can be challenging due to differences in sequencing technology, how MGD is defined, variant evaluation protocols, and reporting criteria. Technical advantage of genome sequencing over exome sequencing in improving the resolution of copy number variant detection has been discussed in previous literatures [8, 35, 36]. Specifically, GS in patient PKIG01887 (Table 3 and Supplementary Table 5), whose previous ES was nondiagnostic, identified additional variants in two genes. These variants include a single exon deletion and a missense variant. While we would expect at least the missense variant to be identified by ES, technical differences between ES and GS sample preparation can produce discordant sequencing results. ES requires PCR amplification directed by an extensive probe set, which can introduce amplification bias or allele dropout. The PCR-free simplicity of GS provides the most comprehensive exome via even, consistent coverage of disease-associated intergenic regions of the genome. Moreover, the definition of MGD requires careful consideration. In our analysis, cases with terminal losses and gains suggestive of derivative chromosomes from parental balanced translocations were counted as a single diagnosis. However, both DMGD and PMGD were grouped as MGD. In PMGD cases, a single P/LP variant coexists with a VUS that accounts for additional, distinct phenotypes not explained by the P/LP variant. Although the clinical significance of the variant was uncertain, strong clinical relevance justified inclusion in our diagnostic yield. This approach is supported by the potential for reclassification of the VUS as our understanding of gene-disease relationships evolves. Despite these nuances, the notable prevalence of MGD underscores the importance of multidisciplinary investigation, especially when a confirmed, single genetic diagnosis does not explain the entirety of the clinical presentation. In addition to the MGD cases, 9 cases feature at least one definitive diagnostic and one secondary finding. Although not classified as multiple diagnoses, these cases carry significant clinical and ethical implications, particularly in the areas of long-term healthcare and reproductive planning.

An in-depth review, exemplified by case PKIG00063, enriches our understanding of the complex landscape of pediatric genetic disorders and further highlights the crucial role of GS in deciphering intricate genetic architectures beyond initial impressions. The detection of uniparental isodisomy and the likely pathogenic variant in the *ERCC8* gene in addition to the previously identified trisomy 21 and copy number gain of 2q11.1q12.1 highlight the challenges in achieving nuanced diagnostic accuracy, thereby refining the quality of genetic counseling and family planning recommendations. Similarly, in case PKIG01276 (Table 2 and Supplementary Table 4), an initial microarray identified a 17p12 duplication associated with Charcot-Marie-Tooth disease but did not explain the patient's intellectual disability and global developmental delay. Subsequent GS revealed an additional pathogenic SNV in *POGZ*, associated with White-Sutton syndrome, and a 15q11.2 deletion overlooked by the initial microarray. Although ACMG guidelines now recommend GS or ES for pediatric patients with congenital anomalies or intellectual disability [37], microarrays persist due to reimbursement challenges. If the 15q11.2 deletion had been initially identified, would the genetic clinic have proceeded with GS that revealed White-Sutton syndrome? These instances are not isolated occurrences. In 19 MGD cases with prior testing, 17 had inconclusive or partial results. Luckily, these cases all exhibited phenotypes that led physicians to explore additional underlying causes. This emphasizes the necessity for a thorough and inclusive genetic testing approach, even with an initial diagnosis. Specifically, clinicians should scrutinize whether initial diagnostic findings comprehensively account for the observed phenotypes to ensure optimal therapeutic strategies and management.

The observed diversity in phenotypic presentation of those with MGD—distinctive in 17, overlapping in 9, and mixed in 2—adds another layer of diagnostic complexity. This heterogeneity directly challenges traditional diagnostic approaches that employ targeted gene panels for phenotype-based hypotheses [2, 4, 38]. For instance, in PKIG00155, karyotyping and *FGFR3* gene sequencing returned inconclusive results. Comprehensive GS, on the other hand, revealed crucial variants in *COL2A1* and *PCDH19* that clarified the patient's complex clinical picture. Such diagnostic limitations are exacerbated in cases with overlapping or extended phenotypes; clinicians may halt further investigation once a diagnosis is obtained through panel or single-gene testing, potentially resulting in missed diagnoses [3]. Therefore, our findings underscore the imperative for versatile and robust diagnostic methodologies to effectively navigate the intricate landscape of multiple genetic diagnoses.

In alignment with existing research on the clinical utility of GS in neonatal intensive care units, our study emphasizes its effectiveness in diagnosing MGDs. Our data demonstrate an overall diagnostic yield of 30% in infants including 25% in SDD and 5% in MGD. This is consistent with the previous studies on pediatric and acutely ill infants, with a diagnostic yield ranging from 34% to 43% [8, 39–41]. Our data first demonstrated that neonates diagnosed with MGD accounted for a remarkable 4.9% of the total infant cohort and 16.7% of all diagnosed infant cases. Intriguingly, this is substantially higher than in older age groups, where diagnostic yields ranged between 1% and 2% of the corresponding age cohort. This elevated incidence in neonates could be attributable to increased vulnerability to multisystemic genetic disorders [42, 43], stressing the urgent need to broaden the use of GS in neonatal settings for the accurate and timely diagnosis of MGDs.

Of note, we acknowledge certain limitations in this study. Our GS techniques are not optimized for detection of balanced or complex structural variants, which have known pathological implications. Second, in instances where external genetic testing had been previously conducted, we were unable to perform methodological comparisons or ascertain the reasons for discrepancies in findings. Finally, we did not include reanalysis of existing data, even though current literature indicates that such reanalysis could identify additional diagnostic findings [44]. Consequently, the actual MGD yield may exceed the number stated in this study.

## 5. Conclusions

Our findings and presented case studies extend our understanding of the prevalence of MGD among pediatric patients with suspected Mendelian disorders. They not only illuminate the complex terrain of rare disease but also accentuate the vital role of a comprehensive, genome-level diagnostic approach.

## Figures and Tables

**Table 1:** Demographic characteristics of all pediatric GS cases.

	Total no. (%)	Neonates (<1 M)	Infants (1-12 M)	Toddlers (13-36 M)	Children (3-11 Y)	Adolescents (12-18 Y)
All cases	1487 (100)	61 (4.1)	241 (16.2)	233 (15.7)	657 (44.2)	295 (19.8)
Proband sex						
Female	647 (43.5)	22 (3.4)	121 (18.7)	105 (16.2)	272 (42.0)	127 (19.6)
Male	840 (56.5)	39 (4.6)	120 (14.3)	128 (15.2)	385 (45.8)	168 (20.0)
Previous testing						
With prior genetic testing	694 (46.7)	11 (1.6)	88 (12.7)	122 (17.6)	330 (47.6)	143 (20.6)
Without prior genetic testing	793 (53.3)	50 (6.3)	153 (19.3)	111 (14.0)	327 (41.2)	152 (19.2)
Sample type						
DBS	200 (13.4)	12 (6.0)	49 (24.5)	42 (21.0)	79 (39.5)	18 (9.0)
Saliva	632 (42.5)	6 (0.9)	53 (8.4)	97 (15.3)	309 (48.9)	167 (26.4)
Whole blood	461 (31.0)	38 (8.2)	112 (24.3)	63 (13.7)	173 (37.5)	75 (16.3)
gDNA	194 (13.0)	5 (2.6)	27 (13.9)	31 (16.0)	96 (49.5)	35 (18.0)
Analysis						
Singleton	711 (47.8)	35 (4.9)	135 (19.0)	114 (16.0)	293 (41.2)	134 (18.8)
Trio	776 (52.2)	26 (3.4)	106 (13.7)	119 (15.3)	364 (46.9)	161 (20.7)
Secondary findings						
ACMG	1145 (77.0)	33 (2.9)	171 (14.9)	181 (15.8)	526 (45.9)	234 (20.4)
Other diagnostic	869 (58.4)	24 (2.8)	113 (13.08)	129 (14.8)	425 (48.9)	178 (20.5)
Carrier status	884 (59.4)	24 (2.7)	110 (12.4)	132 (14.9)	436 (49.3)	182 (20.6)
PGx	881 (59.2)	23 (2.6)	106 (12.0)	141 (16.0)	425 (48.2)	186 (21.1)

M: months; Y: years; CI: confidence interval; DBS: dried blood spots; gDNA: genomic DNA; ACMG: American College of Medical Genetics and Genomics; PGx: pharmacogenomics.

**Table 2:** Genotype-phenotype correlation in cases positive for definitive multiple genetic diagnoses.

Study ID	Submitted clinical presentation	Prior genetic testing	Molecular mechanism	Dx/related features	Overlapping/distinctive
PKIG00155	3-year-old female with short stature, brachydactyly, macrocephaly, progressive lumbar kyphosis, very short extremities, shortening of tubular bones of both extremities appears with widening and lower lumbar lordosis, febrile seizures, concern for achondroplasia and hypochondroplasia	External karyotype and panel testing: nondiagnostic	*COL2A1* c.2257G>A p.(Gly753Ser), heterozygous, pathogenic	*COL2A1*-related skeletal dysplasia/short stature, brachydactyly, macrocephaly, progressive lumbar kyphosis, very short extremities, shortening of tubular bones of both extremities appears with widening and lower lumbar lordosis	Distinctive
			*PCDH19* c.2571_2572delGC p.?, heterozygous, pathogenic	Developmental and epileptic encephalopathy 9/febrile seizures	

PKIG00180	1-month-old female with nonimmune fetal hydrops, dysmorphic facial features (low-set ears and upslanting palpebral fissure) concerning for Down syndrome, skeletal dysplasia, Noonan syndrome, severe edema/ascites, hypotension, hypothyroidism, elevated amino acid, SCID, large perimembranous VSD, persistent pulmonary hypertension of newborn, single palmar crease on left hand, rhizomelic shortening of upper and lower extremities, polyhydramnios, premature birth	None	33.7 Mb dup of 21q11.2q22.3, pathogenic	Down syndrome/dysmorphic facial features, severe edema/ascites, hypotension, hypothyroidism, large perimembranous VSD, single palmar crease on left hand, rhizomelic shortening of upper and lower extremities	Distinctive
			*GLA* c.679C>T p.(Arg227Ter), heterozygous, pathogenic	Fabry disease, cardiac variant/too early to manifest	

PKIG00282 [7]	1.5-month-old male with sudden death, IUGR, small for gestational age, congenital cardiac malformation, right ventricular hypertrophy, bilateral peripheral pulmonary artery stenosis, patent foramen ovale, thickened aortic valve, aortic arch hypoplasia, arrhythmia, hypertension, Williams syndrome, hypoglycemia, hepatomegaly, dysmorphic facial features, inborn error of metabolism	External CMA: 7q11.23 deletion reported	1.5 Mb del of 7q11.23, pathogenic	Williams-Beuren syndrome/IUGR, small for gestational age, congenital cardiac malformation, bilateral peripheral pulmonary artery stenosis, patent foramen ovale, thickened aortic valve, aortic arch hypoplasia, dysmorphic facial features	Distinctive
			1.04 Mb dup of 16p13.11, likely pathogenic	16p13.11 microduplication syndrome/right ventricular hypertrophy, arrhythmia, hypertension	

PKIG00374 [7]	17-year-old male with bilateral optic nerve colobomata, bifid uvula, seizures, focal epilepsy onset at 1 year old, bilateral ptosis, congenital nystagmus, myopia, microphthalmia, cryptorchidism, developmental delay, nonverbal, severe speech and language delay, intellectual disability, legal blindness, palmoplantar hyperkeratosis, dolichocephaly, long face, low-set ears, tapering fingers, hyperpigmentation on lower right leg, bilateral sensorineural hearing loss	External karyotype and panel testing: nondiagnostic External CMA: 9q22.32 duplication reported	*PACS1* c.607C>T p.(Arg203Trp), heterozygous, pathogenic	Schuurs-Hoeijmakers syndrome/focal epilepsy, bilateral ptosis, congenital nystagmus, myopia, developmental delay, nonverbal, severe speech and language delay, intellectual disability, low-set ears	Distinctive
			*GJB2* c.101T>C p.(Met34Thr), heterozygous, pathogenic	*GJB2*-related deafness/bilateral sensorineural hearing loss, palmoplantar hyperkeratosis	
			*GJB2* c.109G>A p.(Val37Ile), heterozygous, pathogenic		

PKIG00405^∗^	6-year-old female with dysgenesis of corpus callosum, muscular hypotonia, microcephaly, delayed motor development, delayed language development, intellectual disability, attention deficit disorder, elevated liver enzymes, coloboma, microphthalmia, strabismus, visual impairment, hypodontia, dysmorphic facial features, hypotelorism, upslanting palpebral fissure, low-set protruding posterior rotated ears, midface hypoplasia, prominent everted upper lips, small chin, small nails, clinodactyly, overlapping toes, failure to thrive, IUGR, oligohydramnios. Clinical features manifested in infancy	External karyotype: nondiagnostic	*THOC6* c.810+1G>A p.?, homozygous, likely pathogenic	Beaulieu-Boycott-Innes syndrome/microcephaly, delayed motor development, delayed language development, intellectual disability, dysmorphic facial features, upslanting palpebral fissure, prominent everted upper lips	Distinctive, overlapping feature: visual impairment
			*ABCA4* c.5882G>A p.(Gly1961Glu), homozygous, pathogenic with reduced penetrance	*ABCA4*-related ocular disorders; retinitis pigmentosa 1/visual impairment	
			*RP1* c.5248G>T p.(Glu1750Ter), homozygous, likely pathogenic		

PKIG00596	5-year-old male born in a consanguineous couple presented with delayed motor development, delayed language development, developmental delay, hypertension, microcornea, ptosis, sclerocornea, bilateral symmetrical globi pallidi hypodensities, hyperkalemia, poor growth	External panel testing: *MMUT* variant reported	*MMUT* c.2200C>T p.(Gln734Ter), homozygous, pathogenic	Methylmalonic aciduria, mut(0) type/delayed motor development, delayed language development, developmental delay, bilateral symmetrical globi pallidi hypodensities, poor growth	Distinctive
			*KERA* c.1026del p.?, homozygous, likely pathogenic	Cornea plana 2/microcornea, sclerocornea	

PKIG01041	5-year-old female with developmental delay, congenital hypotonia, right side weakness	None	*CUL3* c.264+1G>C p.?, heterozygous, likely pathogenic	Neurodevelopmental disorder with or without autism or seizures/developmental delay	Overlapping feature: developmental delay
			1.5 Mb dup of 22q11.21, pathogenic	Chromosome 22q11.2 duplication syndrome/developmental delay, congenital hypotonia, right side weakness	
			836.3 kb dup of 22q11.21, pathogenic		

PKIG01194	1-week-old female with craniosynostosis, scleroderma, visual impairment, multiple joint contractures, syndactyly, talipes equinovarus, defect of ventricular septum, thirteen pairs of ribs, early ossification of femoral heads, narrow chest, narrow palpebral fissure, corneal opacities, macroglossia, ear anomalies, micrognathia	None	3.5 Mb del of 2q37.3, pathogenic	Unbalanced translocation involving chromosomes 2q and 9q/syndactyly, talipes equinovarus, thirteen pairs of ribs, early ossification of femoral heads, narrow chest, narrow palpebral fissure, macroglossia, ear anomalies, micrognathia, craniosynostosis	Distinctive
			17.7 Mb dup of 9q33.2q34.3, pathogenic		
			1.3 Mb dup of 16p13.11, likely pathogenic	Chromosome 16p13.11 microduplication syndrome/too early to manifest	

PKIG01276^∗^	5-year-old male with Charcot-Marie-Tooth disease, developmental delay starting at 9 month old, intellectual disability, femoral congenital anteversion, craniosynostosis, facial hypotonia, broad and flat nasal bridge, telecanthus	External CMA: 17p12 duplication and 18q12.2 deletion (likely benign) reported	1.4 Mb dup of 17p12, pathogenic	Charcot Marie-Tooth disease Type 1A/Charcot-Marie-Tooth disease	Distinctive, overlapping features: developmental delay, intellectual disability
			*POGZ* c.2324_2325insTT p.?, heterozygous, likely pathogenic	White-Sutton syndrome/developmental delay, intellectual disability, facial hypotonia, broad and flat nasal bridge, telecanthus; 15q11.2 deletion syndrome/developmental delay, intellectual disability	
			529.4 kb del of 15q11.2, pathogenic		

PKIG01425	8-year-old male with short stature, pulmonary valve stenosis, supravalvular pulmonic stenosis, glycogen storage disease, eosinophilic esophagitis. Clinical features manifested around 6 weeks	External *G6PC* sequencing: *G6PC* variant reported	*PTPN11* c.1492C>T p.(Arg498Trp), heterozygous, pathogenic	Noonan spectrum disorder/short stature, pulmonary valve stenosis, supravalvular pulmonic stenosis	Distinctive
			*G6PC* c.247C>T p.(Arg83Cys), homozygous, pathogenic	Glycogen storage disease Ia/short stature, glycogen storage disease	

PKIG01849	2-month-old male with microcephaly, hypertonia, small for gestational age, slow growth, IUGR, failure to thrive, underdeveloped inferior vermis, cryptorchidism, low-set posteriorly rotated ears, poor feeding, gastroesophageal reflux, aortic root dilation, phenylketonuria	External CMA: nondiagnostic	*KAT6A* c.3385C>T p.(Arg1129Ter), heterozygous, pathogenic	Arboleda-Tham syndrome/microcephaly, small for gestational age, slow growth, IUGR, failure to thrive, low-set posteriorly rotated ears, poor feeding, gastroesophageal reflux	Distinctive
			*PAH* c.527G>T p.(Arg176Leu), heterozygous, pathogenic	Phenylketonuria/microcephaly, phenylketonuria	
			*PAH* c.898G>T p.(Ala300Ser), heterozygous, pathogenic		

PKIG02092	2-year-old male with failure to thrive, gross motor developmental delay, speech delay, global developmental delay, congenital hypotonia, congenital nystagmus, esotropia, hypospadias, cryptorchidism, feeding difficulties, dysphagia, neurofibromatosis type 1. Clinical features manifested at 8 months	*NF1* and *SPREAD1* sequencing: *NF1* variant reported	11.9 Mb dup of 20q13.2q13.33, pathogenic	20q trisomy/gross motor developmental delay, speech delay, global developmental delay	Overlapping feature: developmental delay
			11.9 Mb dup of 13q11q12.3, pathogenic	13q trisomy/failure to thrive, gross motor developmental delay, speech delay, global developmental delay	

PKIG02101	1-week-old male with arrhythmia, respiratory failure, possible lactic acidosis, seizures, apnea, neonatal jaundice, hyperkalemia, hypomagnesemia, transitory hyperammonemia, deformity of feet, anuria, oliguria, abnormal kidney function, abnormal phosphorus metabolism, abnormal liver function, hypotension	None	600.5 kb del of Xp11.4, hemizygous, pathogenic	Ornithine transcarbamylase deficiency/seizures, apnea, hyperammonemia	Distinctive
			*RORB* c.286G>T p.(Glu96Ter), heterozygous, likely pathogenic	Susceptibility to idiopathic generalized epilepsy 15/seizures	

PKIG00063^∗^	11-year-old male presented seizure with clonic movement, oligohydramnios, IUGR, microphthalmia, bilateral congenital cataract, nonpalpable testes, multiple VSD with Gerbode defect, enlarged epiglottis, laryngomalacia, tracheomalacia, failure to thrive, respiratory distress, asthma, adrenal insufficiency, hypothyroidism, gonadotropin deficiency, microphallus, sleep apnea, sensorineural hearing loss, diabetic ketoacidosis, left hemiparesis, nephrotic syndrome, development delay, hypotonia, congenital heart defects, hyperglycemia. Clinical features present at birth	External karyotype: trisomy 21 reported External CMA: trisomy 21, 2q11.1q12.1 duplication, and 5p15.33q35.3 AOH reported	*ERCC8* c.613G>C p.(Ala205Pro), homozygous, likely pathogenic	Cockayne syndrome, type A/seizure, IUGR, cataract, hypogonadism, sensorineural hearing loss, left hemiparesis, hypotonia	Overlapping features: seizure, hearing loss, hypotonia
			8.8 Mb dup of 2q11.1q12.1, likely pathogenic	Sensorineural hearing loss, seizure/VSD, congenital heart defects, seizure, hypotonia, hypothyroidism, gonadotropin deficiency	
			Trisomy 21, pathogenic		

PKIG00099	11-year-old male with autism, Asperger's, ADHD, abnormal myelination, frontal lobe demyelination, ataxia, visual impairment, blurry vision, eye pain, abnormal VEP, bilateral sensorineural hearing loss, hyporeflexia, neuropathy, clinical improvement (less ataxia, return to reflexes) with high-dose riboflavin (B2) supplementation, constipation. Clinical features manifested at 10 years	External aCGH/SNP analysis: XXY aneuploidy reported	*ACOX1* c.710A>G p.(Asn237Ser), heterozygous, likely pathogenic	Mitchell syndrome/abnormal myelination, frontal lobe demyelination, ataxia, hearing loss, hyporeflexia, neuropathy	Distinctive
			XYY aneuploidy pathogenic	Jacobs syndrome/ADHD	

^∗^2nd mechanism and 2nd Dx include two molecular diagnoses with overlapping clinical presentations. Abbreviations: Dx: diagnosis; SCID: severe combined immunodeficiency; VSD: ventricular septal defect; IUGR: intrauterine growth restriction; ADHD: attention deficient hyperactivity disorder; VEP: visual evoked potential; CMA: chromosomal microarray; aCGH: array comparative genomic hybridization SNP: single nucleotide polymorphism; VUS: variant of uncertain significance. Note: for comprehensive case-level information, including HPO terms, variant nomenclature, and other details, please refer to Table S4 in the supplement.

**Table 3:** Genotype-phenotype correlation in cases positive for presumed multiple genetic diagnoses.

Study ID	Submitted clinical presentation	Prior genetic testing	Molecular mechanism	Dx/related features	Overlapping/distinctive
PKIG00165 [7]	9-month-old male with severe growth and developmental delay, failure to thrive, Romano-Ward syndrome, hx of G-tube feeding, metabolic disorder, mitochondrial metabolic disorder, agenesis of corpus callosum, seizure disorder, hypertension, irregular breathing pattern, hypoxia, lactic acidosis, acute respiratory failure with hypoxia and hypercapnia, respiratory distress, respiratory insufficiency, half-sibling with autism	External WES: *KCNH2* variant and *SLC25A19* variants reported	*KCNH2* c.774_789del p.?, heterozygous, pathogenic	Long QT syndrome 2; short QT syndrome 1/Romano-Ward syndrome	Distinctive
			*SLC25A19* c.115A>T p.(Ile39Phe), heterozygous, VUS	Microcephaly, Amish type; thiamine metabolism dysfunction syndrome 4/severe growth and developmental delay, lactic acidosis	
			*SLC25A19* c.433C>A p.(Arg145Ser), heterozygous, VUS		

PKIG00217	8-year-old-female with mixed conductive and sensorineural hearing loss, short stature, bilateral hypermetropia, bilateral 5th finger brachydactyly, bilateral little finger clinodactyly, hoarseness. Clinical features manifested at 6 months	External panel testing: *MYO3A* VUS reported	*ANKRD11* c.2288_2289del p.?, heterozygous, pathogenic	KBG syndrome/short stature, bilateral 5th finger brachydactyly, bilateral little finger clinodactyly	Distinctive
			*TNC* c.5692G>A p.(Val1898Ile), heterozygous, VUS	Deafness 56/hearing loss	

PKIG00252	14-year-old male with small pituitary gland, delayed motor development, delayed language development, nonverbal, profound intellectual disability, chorea, congenital muscular hypotonia, focal seizure, chronic encephalopathy, esotropia, inguinal hernia, self-injurious behavior. Clinical features manifested around 5 months	External karyotype, aCGH, myotonic dystrophy testing, Angelman/Prader-Willi syndrome methylation analysis, fragile X testing: nondiagnostic	*CACNA1A* c.2042_2043del p.?, heterozygous, pathogenic	Early infantile epileptic encephalopathy 42; episodic ataxia, type 2; familial hemiplegic migraine 1; spinocerebellar ataxia 6/delayed motor development, delayed language development, profound intellectual disability, chorea, hypotonia, seizure, encephalopathy	Overlapping features: delayed motor development, delayed language development, profound intellectual disability, hypotonia, seizure, encephalopathy
			*GABBR2* c.1724C>A p.(Thr575Asn), heterozygous, VUS	Early infantile epileptic encephalopathy 59; neurodevelopmental disorder with poor language and loss of hand skills/delayed motor development, delayed language development, nonverbal, profound intellectual disability, hypotonia, seizure, encephalopathy, self-injurious behavior	

PKIG00301	7-year-old female with delayed motor development, delayed language development, intellectual disability, ataxia, muscular hypotonia, microcephaly, blepharospasm, ptosis, visual impairment, external ear malformation, postaxial polydactyly in hands, constipation, failure to thrive, short stature, congenital malformation of face and neck, aphasia. Clinical features manifested at 2 months	External karyotype: 3p25.3p26.1 deletion and 12p13.31 deletion of unknown significance reported	1.9 Mb del of 3p26.1p25.2, heterozygous, pathogenic	3p- syndrome/delayed motor development, delayed language development, intellectual disability, microcephaly, ptosis, hypotonia, postaxial polydactyly	Overlapping feature: delayed motor development, delayed language development, intellectual disability, microcephaly, hypotonia
			*GRIN2B* c.1427A>G p.(Tyr476Cys), heterozygous, VUS	Developmental and epileptic encephalopathy 27; intellectual developmental disorder 6 with or without seizures/delayed motor development, delayed language development, intellectual disability, microcephaly, hypotonia	

PKIG00439	18-year-old male with immunodeficiency, schizophrenia, dystonia, stroke like symptoms, ADHD, developmental delay, developmental regression, intellectual disability, ataxia, muscular hypotonia, encephalopathy, headache/migraine, abnormal creatine kinase, hyperalaninemia, increased serum pyruvate, ketosis, visual impairment, abnormal skeletal system, abnormal vertebral column, joint hypermobility, scoliosis, abnormal nail, constipation, failure to thrive, vomiting, polyhydramnios. Clinical features manifested at 3.5 years and schizophrenia manifested at 18 years	None	*GATA2* c.1017+572C>T p.?, heterozygous, pathogenic	Emberger syndrome; immunodeficiency 21/immunodeficiency	Distinctive
			*KDM6B* c.1069G>A p.(Gly357Arg), heterozygous, VUS	Neurodevelopmental disorder with coarse facies and mild distal skeletal abnormalities/developmental delay, ADHD, developmental regression, intellectual disability, hypotonia, joint hypermobility	

PKIG00483	6-year-old male with intellectual disability, large stem, abnormal legs and hands, speech problem	None	188.8 kb intragenic del of *DYM* exons 6-13, heterozygous, pathogenic	Dyggve-Melchior-Clausen disease; Smith-McCort dysplasia/large stem, abnormal legs and hands	Distinctive
			5.1 kb intragenic del of *DYM* exon 13, homozygous, pathogenic		
			*PTCHD1* c.1636C>G p.(Gln546Glu), hemizygous, VUS	Susceptibility to autism 4/intellectual disability, speech problem	

PKIG01019	1-month-old female with multiple congenital anomalies, pyelectasis, cataract, coloboma, microphthalmia, visual impairment, high arched palate, hyperbilirubinemia, neonatal jaundice, hydronephrosis, abnormal renal morphology, bilateral single transverse palmar creases, respiratory insufficiency, aberrant right coronary artery, suspected Alagille syndrome	None	11.7 Mb del of 14q31.2q32.12, heterozygous, pathogenic	Bilateral single transverse palmar creases	Distinctive
			*RAB23* c.-66+4614dup p.?, heterozygous, VUS	Carpenter syndrome/visual impairment, high arched palate, pyelectasis	
			*RAB23* c.434T>A p.(Leu145Ter), heterozygous, pathogenic		

PKIG01287 [7]	3-year-old female with ataxia, seizure, encephalopathy, hypertelorism, flat nasal bridge, wide nasal tip; differentials include spinocerebellar ataxia, ataxia telangiectasia, Friedreich ataxia, episodic ataxia, mitochondrial disorders, and inborn errors of metabolism (organic acidemia, urea cycle defect, maple syrup urine disease, CoQ10 deficiency). Clinical features manifested at 35 months	None	1.5 Mb dup of 16p13.10, likely pathogenic	16p13.11 microduplication syndrome/seizure	Distinctive
			PC c.2000A>G p.(Lys667Arg), homozygous, VUS	Pyruvate carboxylase deficiency/seizure, encephalopathy	

PKIG01703	16-month-old female with failure to thrive, hypotonia, global developmental delay, constipation, trigonocephaly, bitemporal narrowing, short nose, anteverted nares, simplified philtrum, low-set ears, joint hypermobility	External CMA, hypotonia panel testing (incl. myotonic dystrophy, SMA, and Prader-Willi syndrome), and fragile X testing: nondiagnostic	*PPM1D* c.1224dup p.?, heterozygous, likely pathogenic	Jansen de Vries syndrome/failure to thrive, hypotonia, global developmental delay, constipation, anteverted nares, low-set ears	Distinctive
			*SCAPER* c.1382A>T p.(Asp461Val), heterozygous, VUS	Intellectual developmental disorder and retinitis pigmentosa/global developmental delay	
			*SCAPER* c.1250C>T p.(Ser417Phe), heterozygous, VUS		

PKIG01847	6-year-old male with Pierre Robin sequence, spasticity, spastic diplegic cerebral palsy, developmental delay, arthrogryposis of lower extremities, redundant nuchal skin, bilateral transverse palmar crease, hair tuft on lower back, tethered spinal cord, focal epilepsy, congenital velopharyngeal insufficiency, toe-walking, myalgias, self-injurious behavior, sleep disturbance, short stature, attention deficit hyperactivity disorder. Clinical features present at birth	External CMA: nondiagnostic External Stickler syndrome panel testing: *COL11A1* VUS reported	*WAC* c.265_266del p.?, heterozygous, pathogenic	Desanto-Shinawi syndrome/developmental delay, epilepsy, attention deficit hyperactivity disorder, self-injurious behavior	Distinctive
			11.9 kb intragenic del of *PIEZO2* intron 2, heterozygous, VUS	Distal arthrogryposis type 3; distal arthrogryposis type 5; distal arthrogryposis with impaired proprioception and touch/arthrogryposis of lower extremities, Pierre Robin sequence, developmental delay, epilepsy, bilateral transverse palmar crease	

PKIG01887 [7]	3-year-old female with global developmental delay, delayed motor development, delayed language development, developmental regression, intellectual disability, muscular hypotonia, spasticity, epileptic activity, microcephaly, difficulty chewing, difficulty swallowing, poor sucking, severe constipation, gastroesophageal reflux disease, allergies, behavioral issues, grinding, hand biting, repetitive behavior, hyperopia, ankyloglossia. Clinical features manifested at 9 months	External WES: *G6PD* variant unrelated to phenotype reported	24.0 kb intragenic del of *MECP2* exon 3, heterozygous, pathogenic	Neonatal severe encephalopathy; syndromic intellectual developmental disorder 13; syndromic intellectual developmental disorder, Lubs type; Rett syndrome/global developmental delay, delayed motor development, delayed language development, developmental regression, intellectual disability, muscular hypotonia, spasticity, epileptic activity, microcephaly, difficulty chewing, difficulty swallowing, poor sucking, severe constipation, Gastroesophageal reflux disease, behavioral issues, grinding, hand biting, repetitive behavior	Overlapping features: global developmental delay, delayed motor development, delayed language development, developmental regression, intellectual disability, muscular hypotonia, spasticity, epileptic activity, microcephaly
			*DNM1* c.352G>C p.(Val118Leu), heterozygous, VUS	Developmental and epileptic encephalopathy 31A; developmental and epileptic encephalopathy 31B/global developmental delay, delayed motor development, delayed language development, developmental regression, intellectual disability, muscular hypotonia, spasticity, epileptic activity, microcephaly	

PKIG02037	6-year-old female with focal epilepsy and epileptic syndromes with simple partial seizures, intractable, without status epilepticus, refractory epilepsy, autistic disorder, gray matter and periventricular heterotopia, absent speech, intellectual disability, global developmental delay, optic nerve dysplasia, cortical visual impairment, neurogenic bladder cleft palate, congenital hypotonia, conductive hearing loss, recurrent infections, motor stereotypies, tethered cord syndrome, visual-spatial disorder, toe walking. Clinical features manifested in infancy	External aCGH, Angelman panel test: nondiagnostic External mtDNA: *MT-TL* variant reported External WES: *DMXL2* variants and *SHANK3* variant reported	*MT-TL1* m.3243A>G, heteroplasmic, pathogenic	Mitochondrial encephalomyopathy, lactic acidosis and stroke-like episodes (MELAS)/epilepsy, hypotonia	Distinctive, overlapping features: epilepsy, hypotonia
			*DMXL2* c.7268G>T p.(Arg2423Ile), heterozygous, VUS	Developmental and epileptic encephalopathy 81/epilepsy, absent speech, intellectual disability, global developmental delay, hypotonia	
			*DMXL2* c.5974G>A p.(Asp1992Asn), heterozygous, VUS		

PKIG02059	2-year-old male with developmental delay, speech regression, gross motor delay, hypotonia, neutropenia, dysphagia for liquids, supraventricular tachycardia, possible hypothyroidism, progressive sleepiness. Cardiology phenotypes manifested at 1 month	None	*COQ8A* c.1651G>A p.(Glu551Lys), heterozygous, likely pathogenic	Primary coenzyme Q10 deficiency 4/developmental delay, speech regression, gross motor delay, hypotonia	Overlapping features: developmental delay, speech regression, gross motor delay, hypotonia
			*COQ8A* c.901C>T p.(Arg301Trp), heterozygous, likely pathogenic		
			*CIC* c.298G>A p.(Ala100Thr), heterozygous, VUS	Intellectual developmental disorder 45/developmental delay, speech regression, gross motor delay, hypotonia	

Abbreviations: Dx: diagnosis; ADHD: attention deficient hyperactivity disorder; CMA: chromosomal microarray; aCGH: array comparative genomic hybridization; SNP: single nucleotide polymorphism; VUS: variant of uncertain significance. Note: for comprehensive case-level information, including HPO terms, variant nomenclature, and other details, please refer to Table S5 in the supplement.

## Data Availability

Access to deidentified data that are not provided may be requested via the corresponding author.

## Supplementary Materials

Supplementary Materials Table S1: genes/loci associated with known repeat expansion disorders. Table S2: genes included in the pharmacogenetics secondary finding category. Table S3: genes included in the carrier status secondary findings category. Table S4: GS cases with phenotypic features explained by definitive multiple genetic diagnoses. Table S5: GS cases with phenotypic features explained by presumed multiple genetic diagnoses. Table S6: GS cases with a definitive genetic diagnosis and a reportable secondary finding.
