# Supplementary material for: Beyond Single Diagnosis: Exploring Multidiagnostic Realities in Pediatric Patients through Genome Sequencing
Source: Hum Mutat. 2024 Apr 23;2024:9115364. doi: 10.1155/2024/9115364 (PMC11919036; doi:10.1155/2024/9115364)
Supplement: Supplementary Materials — Table S1: genes/loci associated with known repeat expansion disorders. Table S2: genes included in the pharmacogenetics secondary finding category. Table S3: genes included in the carrier status secondary findings category. Table S4: GS cases with phenotypic features explained by definitive multiple genetic diagnoses. Table S5: GS cases with phenotypic features explained by presumed multiple genetic diagnoses. Table S6: GS cases with a definitive genetic diagnosis and a reportable secondary finding. [file 9115364.f1.pdf]

Table S1. Genes/Loci associated with known repeat expansion disorders

Gene	Disorder	Inheritance	Nucleotide Repeat	WT Repeat Number	Full-Penetrance Pathogenic Repeat Number	Curated PMID
AFF2	Fragile X syndrome, FRAXE type	XLR	CCG	4-39	≥ 200	8334699
AR	Spinal bulbar muscular atrophy	XLR	CAG	≤ 34	≥ 38	20301508, 30006721
ATN1	Dentatorubral-pallidoluysian atrophy	AD	CAG	6-35	≥ 48	20301664
ATXN1	Spinocerebellar ataxia type 1	AD	CAG	6-35	≥ 39	20301363
ATXN2	Spinocerebellar ataxia type 2	AD	CAG	≤ 31	≥ 37	20301452
ATXN3	Spinocerebellar ataxia type 3	AD	CAG	12-44	60 - 87	20301375
ATXN7	Spinocerebellar ataxia type 7	AD	CAG	4-27	≥ 37	20301433
ATXN8OS/ ATXN8	Spinocerebellar ataxia type 8	AD	(CTA)n(CTG)n	15-50	≥ 71	20301446
ATXN10	Spinocerebellar ataxia type 10	AD	ATTCT	10-32	≥800	20301354
C9orf72	Frontotemporal dementia and/or amyotrophic lateral sclerosis	AD	GGGGCC	2-23	≥ 60	25577942
CACNA1A	Spinocerebellar ataxia type 6	AD	CAG	≤ 18	20 - 33	20301319
CNBP	Myotonic dystrophy type 2	AD	CCTG	11-26	≥ 75	20301639
COMP	Pseudoachondroplasia	AD	GAC	5	6	20301660, 9887340
CSTB	Progressive myoclonic epilepsy type 1	AR	CCCCGCCCCGC G	2-3	≥ 30	20301321
DIP2B	Intellectual development disorder, FRA12 type	AD	CGG	6-23	≥ 128	17236128
DMPK	Myotonic dystrophy type 1	AD	CTG	5-34	≥ 50	20301344, 32851192
FMR1	Fragile X syndrome	XLD	CGG	5-44	≥ 200	20301558, 33795824
	Fragile X-associated tremor/ataxia syndrome; Fragile X-associated primary ovarian insufficiency				55-200	
FOXL2	Blepharophimosis, ptosis, and epicanthus inversus syndrome	AD; AR	GCN	14	15 - 27	20301614, 18726931
FXN	Friedreich ataxia	AR	GAA	5-34	≥ 66	20301458
HOXD13	Syndactyly type V; Synpolydactyly 1; Brachydactyly-syndactyly syndrome	AD	GCN	14-15	≥ 22	17236141
NOP56	Spinocerebellar ataxia type 36	AD	GGCCTG	3-14	≥ 650	21683323, 22492559
PABPN1	Oculopharyngeal muscular dystrophy	AD	GCN	≤10	45248	20301305
PHOX2B	Congenital central hypoventilation syndrome	AD	GCN	≤ 20	25-33	20301600
PPP2R2B	Spinocerebellar ataxia type 12	AD	CAG	7-32	≥ 78	20301317, 11761478
RUNX2	Cleidocranial dysplasia	AD	GCN	≤17	20-27	26220009, 9182765
SOX3	Intellectual development disorder with panhypopituitarism	XL	GCN	9-15	22 - 26	12428212, 24346842
TBP	Spinocerebellar ataxia type 17	AD	CAG	25 - 40	≥ 49	20301611
TBX1	Tetralogy of Fallot	AD	GCN	≤15	≥ 25	11748311, 19948535
TCF4	Fuchs endothelial corneal dystrophy	AD	CTG	≤ 39	≥ 40	25168903, 24255041
ZIC2	Holoprosencephaly	AD	GCN	≤15	≥ 25	20301702, 19177455, 20531442
ZIC3	VACTERL	XLR	GCC	≤10	≥ 12	20452998

Abbreviations: AD, autosomal dominant; AR, autosomal recessive; XL, X-linked; XLD, X-linked dominant; XLR, X-linked recessive; VACTERL, vertebral anomalies, anal atresia, cardiac malformations, tracheoesophageal fistula, renal anomalies, and limb anomalies.

Table S2. Genes included in the pharmacogenetics secondary findings category

Gene	Variant	cDNA	Protein	dbSNP rsID	GenBank
CYP2C19	*2	c.681G>A	p.Pro227=	rs4244285	NM_000769.2
	*3	c.636G>A	p.Trp212Ter	rs4986893	NM_000769.2
	*4	c.1A>G	p.Met1?	rs28399504	NM_000769.2
	*6	c.395G>A	p.Arg132Gln	rs56337013	NM_000769.2
	*8	c.358T>C	p.Trp120Arg	rs72552267	NM_000769.2
	*17	c.-806C>T	–	rs72558186	NM_000769.2
	*35	c.332-23A>G	–	rs41291556	NM_000769.2
CYP2C9	*2	c.430C>T	p.Arg144Cys	rs1799853	NM_000771.3
	*3	c.1075A>C	p.Ile359Leu	rs1057910	NM_000771.3
	*5	c.1080C>G	p.Asp360Glu	rs28371686	NM_000771.3
	*6	c.818delA	–	rs9332131	NM_000771.3
	*8	c.449G>A	p.Arg150His	rs7900194	NM_000771.3
	*11	c.1003C>T	p.Arg335Trp	rs28371685	NM_000771.3
CYP2D6	*2	c.886C>T	p.Arg296Cys	rs16947	NM_000106.5
	*3	c.775del		rs35742686	NM_000106.5
	*4	c.506-1G>A		rs3892097	NM_000106.5
	*6	c.454del		rs5030655	NM_000106.5
	*7	c.971A>C	p.His324Pro	rs5030867	NM_000106.5
	*9	c.841_843delAAG		rs5030656	NM_000106.5
	*10	c.100C>T	p.Pro34Ser	rs1065852	NM_000106.5
	*17	c.320C>T	p.Thr107Ile	rs28371706	NM_000106.5
	*41	c.985+39G>A		rs28371725	NM_000106.5
CYP3A5	*6	c.624G>A	p.Lys208=	rs10264272	NM_000777.4
	*7	c.1035dupT		rs41303343	NM_000777.4
SLCO1B1	*37	c.388A>G	p.Asn130Asp	rs2306283	NM_006446.4
	*5	c.521T>C	p.Val174Ala	rs4149056	NM_006446.4
	*15	c.388A>G	p.Asn130Asp	rs2306283	NM_006446.4
		c.521T>C	p.Val174Ala	rs4149056	NM_006446.4
TPMT	*2	c.238G>C	p.Ala80Pro	rs1800462	NM_000367.3
	*3A	c.460G>A	p.Ala154Thr	rs1800460	NM_000367.3
		c.719A>G	p.Tyr240Cys	rs1142345	NM_000367.3
	*3B	c.460G>A	p.Ala154Thr	rs1800460	NM_000367.3
	*3C	c.719A>G	p.Tyr240Cys	rs1142345	NM_000367.3
UGT1A1	*6	c.211G>A	p.Gly71Arg	rs4148323	NM_000463.2
	*28	c.-53_-52TA (7)		rs8175347	NM_000463.2
	*36	c.-53_-52TA (5)		rs8175347	NM_000463.2
	*37	c.-53_-52 (8)		rs8175347	NM_000463.2
NUDT15	*2	c.50_55dup	p.Gly17_Val18dup	rs61746486	NM_018283.4
		c.415C>T	p.Arg139Cys	rs116855232	NM_018283.4
	*3	c.415C>T	p.Arg139Cys	rs116855232	NM_018283.4
	*9	c.50_55del	p.Gly17_Val18del	rs746071566	NM_018283.4

Table S3. Genes included in the carrier status secondary findings category

Gene	Disorder
AAAS	Achalasia-Addisonianism-Alacrima Syndrome
ABCB11	Progressive Familial Intrahepatic Cholestasis
ABCC6	Pseudoxanthoma Elasticum
ABCC8	Familial Hyperinsulinism
ACAD9	Mitochondrial Complex I Deficiency
ACADL	Long Chain Acyl-CoA Dehydrogenase Deficiency
ACADM	Medium Chain Acyl-CoA Dehydrogenase Deficiency
ACADVL	Very Long Chain Acyl-CoA Dehydrogenase Deficiency
ACAT1	Beta-ketothiolase Deficiency
ACOX1	Peroxisomal Acyl-CoA Oxidase dehydrogenase Deficiency
ACSF3	Combined Malonic and Methylmalonic Aciduria
ADA	Adenosine Deaminase Deficiency
ADAMTS2	Ehlers-Danlos Syndrome, Dermatosparaxis Type
ADGRG1	Bilateral Frontoparietal Polymicrogyria
AGA	Aspartylglucosaminuria
AGL	Glycogen Storage Disease, Type III
AGPS	Rhizomelic Chondrodysplasia Punctata, Type 3
AGXT	Primary Hyperoxaluria, Type 1
AIRE	Polyglandular Autoimmune Syndrome, Type 1
ALDH3A2	Sjogren-Larsson Syndrome
ALDH7A1	Pyridoxine-Dependent Epilepsy
ALG6	Congenital Disorder of Glycosylation, Type Ic
ALMS1	Alstrom Syndrom
ALPL	Hypophosphatasia
AMT	Glycine Encephalopathy AMT-Related
AP3B1	Hermansky-Pudlak Syndrome 2
APOB	Hypobetalipoproteinemia
AQP2	Nephrogenic Diabetes Insipidus
AR	Androgen Insensitivity Syndrome
ARG1	Argininemia
ARSA	Metachromatic Leukodystrophy, ARSA-Related
ARSB	Mucopolysaccharidosis Type VI
ASL	Argininosuccinic Aciduria
ASNS	Asparagine Synthetase Deficiency
ASPA	Canavan Disease
ASPM	Primary Microcephaly 5
ASS1	Citrullinemia, Type 1
ATM	Ataxia-Telangiectasia
ATP6V1B1	Renal Tubular Acidosis with Deafness
ATP7A	Menkes Syndrome
ATP7B	Wilson Disease
ATRX	Alpha-Thalassemia Intellectual Disability Syndrome
BBS1	Bardet-Biedl Syndrome 1
BBS10	Bardet-Biedl Syndrome 10
BBS12	Bardet-Biedl Syndrome 12
BBS4	Bardet-Biedl Syndrome 4
BCHE	Pseudocholinesterase Deficiency
BCKDHA	Maple Syrup Urine Disease, Type Ia
BCKDHB	Maple Syrup Urine Disease, Type Ib
BCS1L	GRACILE syndrome
BLM	Bloom Syndrome
BSND	Bartter Syndrome
BTD	Biotinidase Deficiency
BTK	Agammaglobulinemia, X-Linked
CAPN3	Limb-Girdle Muscular Dystrophy, Type 2A
CASQ2	Catecholaminergic Polymorphic Ventricular Tachycardia 2
CBS	Homocystinuria due to Cystathionine Beta-Synthase Deficiency
CDH23	Usher Syndrome, Type 1D
CEP290	Joubert Syndrome 5
CERKL	Retinitis Pigmentosa 26
CFTR	Cystic Fibrosis
CHM	Choroideremia
CHRNE	Congenital Myasthenic Syndrome 4
CIITA	Bare Lymphocyte Syndrome Type II
CLCN1	Myotonia Congenita, Autosomal Recessive
CLN3	Neuronal Ceroid Lipofuscinosis 3
CLN5	Neuronal Ceroid Lipofuscinosis 5
CLN6	Neuronal Ceroid Lipofuscinosis 6
CLN8	Neuronal Ceroid Lipofuscinosis 8
CLRN1	Usher Syndrome, Type 3
CNGB3	Achromatopsia 3
COL27A1	Steel Syndrome
COL4A3	Alport Syndrome, COL4A3-Related
COL4A4	Alport Syndrome, COL4A4-Related
COL4A5	Alport Syndrome, X-Linked
COL7A1	Dystrophic Epidermolysis Bullosa
CPS1	Carbamoyl Phosphate Synthetase I Deficiency
CPT1A	Carnitine Palmitoyltransferase Ia Deficiency
CPT2	Carnitine Palmitoyltransferase II Deficiency
CRB1	Leber Congenital Amaurosis 8
CRPPA	Muscular dystrophy-dystroglycanopathy, type A, 7; C, 7
CTNS	Cystinosis
CTSK	Pycnodysostosis
CYBA	Chronic Granulomatous Disease
CYBB	Chronic Granulomatous Disease, X-Linked
CYP17A1	17-Alpha-Hydroxylase Deficient Congenital Adrenal Hyperplasia
CYP19A1	Aromatase Deficiency
CYP1B1	Primary Congenital Glaucoma 3
CYP27A1	Cerebrotendinous Xanthomatosis
DBT	Maple Syrup Urine Disease, Type II
DCLRE1C	Omenn Syndrome, DCLRE1C-Related
DES	Myofibrillar Myopathy 1
DHCR7	Smith-Lemli-Opitz Syndrome
DHDDS	Retinitis Pigmentosa 59
DKC1	Dyskeratosis Congenita, X-Linked
DLD	Dihydrolipoamide Dehydrogenase Deficiency
DMD	Duchenne/Becker Muscular Dystrophy
DNAH5	Primary Ciliary Dyskinesia 3
DNAI1	Primary Ciliary Dyskinesia 37
DNAI2	Primary Ciliary Dyskinesia 9
DOK7	Congenital Myasthenic Syndrome 10
DPYD	Dihydropyrimidine Dehydrogenase Deficiency
DSP	Dilated Cardiomyopathy with Woolly Hair and Keratoderma; Epidermolysis Bullosa, Lethal Acantholytic; Skin Fragility-Woolly Hair Syndrome
DYSF	Limb-Girdle Muscular Dystrophy, Type 2B
EDA	Hypohidrotic Ectodermal Dysplasia I, X-Linked
EDAR	Hypohidrotic Ectodermal Dysplasia 10B
EIF2B5	Leukoencephalopathy with Vanishing White Matter
ELP1	Familial dysautonomia; Medulloblastoma
EMD	Emery-Dreifuss Muscular Dystrophy 1, X-Linked
ERCC6	Cockayne Syndrome, Type B
ERCC8	Cockayne Syndrome, Type A
ESCO2	Roberts Syndrome
ETFA	Glutaric Acidemia IIA
ETFDH	Glutaric Acidemia IIC
ETHE1	Ethylmalonic Encephalopathy
EVC	Ellis-van Creveld Syndrome, EVC-Related
EVC2	Ellis-van Creveld Syndrome, EVC2-Related
EYS	Retinitis Pigmentosa 25
F11	Factor XI Deficiency
F8	Hemophilia A
F9	Hemophilia B
FAH	Tyrosinemia, Type I
FAM161A	Retinitis Pigmentosa 28
FANCA	Fanconi Anemia, Group A
FANCC	Fanconi Anemia, Group C
FANCG	Fanconi Anemia, Group G
FH	Fumarase Deficiency
FKRP	Limb-Girdle Muscular Dystrophy, Type 2I
FKTN	Walker-Warburg Syndrome FKTN-Related
G6PC1	Glycogen Storage Disease, Type IA
G6PD	Glucose-6-Phosphate Dehydrogenase Deficiency
GAA	Glycogen Storage Disease, Type II
GALC	Krabbe Disease
GALK1	Galactokinase Deficiency
GALT	Galactosemia
GAMT	Guanidinoacetate Methyltransferase Deficiency
GAN	Giant Axonal Neuropathy 1
GBE1	Glycogen Storage Disease IV
GCDH	Glutaric Acidemia I
Gene	Associated Disorders
GFM1	Combined Oxidative Phosphorylation Deficiency 1
GJB1	Charcot-Marie-Tooth Disease with Deafness, X-Linked
GJB2	Non-Syndromic Hearing Loss, GJB2-Related
GJB6	Non-Syndromic Hearing Loss, GJB6-Related
GLA	Fabry Disesae
GLB1	Mucopolysaccharidosis Type IVB
GLDC	Glycine Encephalopathym, GLDC-Related
GLE1	Congenital Arthrogryposis with Anterior Horn Cell Disease
GLUL	Congenital Glutamine Deficency
GNE	Inclusion Body Myopathy 2
GNPTAB	Mucolipidosis II
GNPTG	Mucolipidosis III
GNS	Mucopolysaccharidosis, Type IIID
GP1BA	Bernard-Soulier Syndrome, Type A1
GP9	Bernard-Soulier Syndrome, Type C
GRHPR	Primary Hyperoxaluria Type II
GUCY2D	Leber Congenital Amaurosis 1
HADH	3-Hydroxyacyl-CoA Dehydrogenase Deficiency
HADHA	Long-Chain 3-Hydroxyacyl-CoA Dehydrogenase Deficiency
HAX1	Severe Congenital Neutropenia, 3
HBB	Beta-Hemoglobinopathies
HEXA	Tay-Sachs Disease
HEXB	Sandhoff Disease
HFE	Hemochromatosis Type 2A
HGSNAT	Mucopolysaccharidosis Type IIIC
HJV	Hemochromatosis, type 2A
HLCS	Holocarboxylase Synthetase Deficiency
HMGCL	HMG-CoA Lyase Deficiency
HOGA1	Primary Hyperoxaluria, Type III
HPRT1	Lesch-Nyham Syndrome
HPS1	Hermansky-Pudlak Syndrome 1
HPS3	Hermansky-Pudlak Syndrome 3
HSD17B3	17-Beta Hydroxysteroid Dehydrogenase III
HSD17B4	D-Bifunctional Protein Deficiency
HSD3B2	3-Beta-Hydroxysteroid Dehydrogenase 2 Deficient CongenitalAdrenal Hyperplasia
HYAL1	Mucopolysaccharidosis Type IX
HYLS1	Hydrolethalus Syndrome
IDS	Mucopolysaccharidosis Type II
IDUA	Mucopolysaccharidosis Type I (Hurler Syndrome)
IL2RG	Severe Combined ImmunoDeficiency, X-Linked
IVD	Isovaleric Acidemia
KCNJ11	Congenital Hyperinsulinism
KCNQ1	Jervell and Lange-Nielsen syndrome
L1CAM	Hydrocephalus, L1CAM-Related
LAMA2	LAMA2-Related Muscular Dystrophy
LAMA3	Herlitz Junctional Epidermolysis Bullosa, LAMA3-Related
LAMB3	Herlitz Junctional Epidermolysis Bullosa, LAMB3-Related
LAMC2	Herlitz Junctional Epidermolysis Bullosa, LAMC2-Related
LARGE1	Walker-Warburg Syndrome LARGE1-Related
LCA5	Leber Congenital Amaurosis 5
LDLR	Familial Hypercholesterolemia, LDLR-Related
LDLRAP1	Familial Hypercholesterolemia, LDLRAP1-Related
LHX3	Combined Pituitary Hormone Deficiency 3
LIFR	Stuve-Wiedemann Syndrome
LIPA	Wolman Disease
LIPH	Hypotrichosis 7
LMNA	Charcot-Marie-Tooth Disease, Type 2B1; Emery-Dreifuss Muscular Dystrophy 3; Mandibuloacral dysplasia
LOXHD1	Deafness, Autosomal Recessive 77
LPL	Lipoprotein Lipase Deficiency
LRPPRC	Leigh Syndrome, French Canadian Type
LZTR1	Noonan Syndrome 2
MAN2B1	Alpha-Mannosidosis
MCCC1	3-Methylcrotonyl-CoA Carboxylase 1 Deficiency
MCCC2	3-Methylcrotonyl-CoA Carboxylase 2 Deficiency
MCOLN1	Mucolipidosis IV
MED17	Postnatal Progressive Microcephaly with Seizures and Brain Atrophy
MEFV	Familial Mediterranean Fever
MESP2	Spondylothoracic Dysostosis
MFSD8	Neuronal Ceroid Lipofuscinosis 7
MKKS	Bardet-Biedle Syndrome 6
MKS1	Meckel-Gruber Syndrome 1
MLC1	Megalencephalic Leukoencephalopathy with Subcortical Cysts 1
MMAA	Methylmalonic Aciduria, cb1A Type
MMAB	Methylmalonic Aciduria, cb1B Type
MMACHC	Methylmalonic Aciduria and Homocystinuria, cb1C Type
MMADHC	Methylmalonic Aciduria and Homocystinuria, cb1D Type
MMUT	Methylmalonic Aciduria, Type mut(0)
MPI	Congenital Disorder of Glycosylation, Type Ib
MPL	Congenital Amegakaryocytic Thrombocytopenia
MPV17	Charcot-Marie-Tooth Disease, Axonal, Type 2EE
MTHFR	Homocystinuria due to MTHFR Deficiency
MTM1	Myotubular Myopathy, X-Linked
MTRR	Homocystinuria Type cblE
MTTP	Abetalipoproteinemia
MUTYH	Adenomas, Multiple Colorectal
MYO7A	Usher Syndrome, Type 1B
NAGLU	Mucopolysaccharidosis, Type IIIB
NAGS	N-Acetylglutamate Synthase Deficiency
NBN	Nijmegen Breakage Syndrome
NDRG1	Charcot-Marie-Tooth Disease, Type 4D
NDUFAF5	Mitochondrial Complex I Deficiency, Nuclear Type 16
NDUFS6	Mitochondrial Complex I Deficiency, Nuclear Type 9
NEB	Nemaline Myopathy 2
NPC1	Niemann-Pick Disease, Type C1/D
NPC2	Niemann-Pick Disease, Type C2
NPHP1	Joubert Syndrome 4; Nephronophthisis 1, juvenile; Senior Loken syndrome-1
NPHS1	Nephrotic Syndrome, Type 1
NPHS2	Nephrotic Syndrome, Type 2
NR0B1	X-Linked Congenital Adrenal Hypoplasia
NR2E3	Enhanced S-Cone Syndrome
NTRK1	Familial Dysautonomia, Type II
OAT	Ornithine Aminotransferase Deficiency
OPA3	3-Methylglutaconic Aciduria, Type III
OTC	Ornithine Carbamoyltransferase Deficiency
PAH	Phenylketonuria
PC	Pyruvate Carboxylase Deficiency
PCCA	Propionic Acidemia, PCCA-Related
PCCB	Propionic Acidemia, PCCB-Related
PCDH15	Usher Syndrome, Type 1F
PDHA1	Pyruvate Dehydrogenase E1-Alpha Deficiency
PDHB	Pyruvate Dehydrogenase E1-Beta Deficiency
PEX1	Zellweger Syndrome Disorders, PEX1-Related
PEX10	Zellweger Syndrome Disorders, PEX10-Related
PEX12	Zellweger Syndrome Disorders, PEX12-Related
PEX2	Zellweger Syndrome Disorders, PEX2-Related
PEX6	Zellweger Syndrome Disorders, PEX6-Related
PEX7	Rhizomelic Chondrodysplasia Punctata, Type 1
PFKM	Glycogen Storage Disease VII
PHGDH	Neu-Laxova Syndrome
PKHD1	Autosomal Recessive Polycystic Kidney Disease
PLP1	Spastic Paraplegia 2, X-Linked
PMM2	Congenital Disorder of Glycosylation, Type 1A
POLG	POLG-Related Disorders
POMGNT1	Muscle-Eye-Brain Disease, POMGNT1-Related
PPT1	Neuronal Ceroid Lipofuscinosis 1
PROP1	Combined Pituitary Hormone Deficiency 2
PRPS1	Arts Syndrome
PSAP	Metachromatic Leukodystrophy, PSAP-Related
PTS	6-Pyruvoyl-Tetrahydropterin Synthase (PTPS) Deficiency
PUS1	Mitochondrial Myopathy and Sideroblastic Anemia
PYGM	Glycogen Storage Disease V
RAB23	Carpenter Syndrome
RAG1	Severe Combined ImmunoDeficiency/Omenn Syndrome, RAG1-Related
RAG2	Severe Combined ImmunoDeficiency/Omenn Syndrome, RAG2-Related
RAPSN	Congenital Myasthenic Syndrome 11
RARS2	Pontocerebellar Hypoplasia, Type 6
RDH12	Leber Congenital Amaurosis 13
RMRP	Cartilage-Hair Hypoplasia
RPE65	Leber Congenital Amaurosis 2
RPGRIP1L	Joubert Syndrome 7
RS1	X-Linked Juvenile Retinoschisis 1
RTEL1	Dyskeratosis Congenita
RYR1	Central Core Disease; Minicore Myopathy with External Ophthalmoplegia; Neuromuscular Disease, Congenital with Uniform Type 1 fibers
SACS	Autosomal Recessive Spastic Ataxia of Charlevoix-Sa-Guenay
SAMHD1	Aicardi-Goutières Syndrome 5
SBDS	Shwachman-Diamond Syndrome 1
SEPSECS	Pontocerebellar Hypoplasia, Type 2D
SGCA	Muscular Dystrophy, Limb-Girdle, Autosomal Recessive 3
SGCB	Muscular Dystrophy, Limb-Girdle, Autosomal Recessive 4
SGCD	Muscular Dystrophy, Limb-Girdle, Autosomal Recessive 6
SGCG	Muscular Dystrophy, Limb-Girdle, Autosomal Recessive 5
SGSH	Mucopolysaccharidosis, Type IIIA
SLC12A3	Gitelman Syndrome
SLC12A6	Andermann Syndrome
SLC17A5	Sall Disease
SLC22A5	Carnitine Deficiency
SLC25A13	Citrullinemia, Type 2
SLC25A15	Hyperornithinemia-Hyperammonemia-Homocitrullinuria Syndrome
SLC26A2	Achondrogenesis, Type IB
SLC26A4	Pendred Syndrome
SLC35A3	Arthrogryposis, Intellectual Disability, and Seizure
SLC37A4	Glycogen Storage Disease, Type IB
SLC39A4	Acrodermatitis Enteropathica
SLC3A1	Cystinuria
SLC4A11	Corneal Dystrophy and Perceptive Deafness
SLC6A8	Cerebral Creatine Deficiency Syndrome 1
SLC7A7	Lysinuric Protein Intolerance
SMARCAL1	Schimke Immunoosseous Dysplasia
SMPD1	Niemann-Pick Disease, Type A/B
STAR	Lipoid Congenital Adrenal Hyperplasia
STS	Ichthyosis, X-linked
SUMF1	Multiple Sulfatase Deficiency
TAT	Tyrosinemia, Type II
TCIRG1	Infantile Malignant Osteopertosis 1
TECPR2	Spastic Paraplegia 49
TFR2	Hemochromatosis Type 3
TGM1	Lamellar Ichthyosis 1
TH	Segawa Syndrome
TMEM216	Joubert Syndrome 2
TMEM67	Joubert Syndrome 6/ Meckel Syndrome 3
TPP1	Neuronal Ceroid Lipofuscinosis 2
TRDN	Cardiac Arrhythmia Syndrome, with or without Skeletal Muscle Weakness
TRMU	Liver Failure, Infantile, Transient (LFIT)
TSEN54	Pontocerebellar Hypoplasia, Type 2A/4
TSFM	Combined Oxidative Phosphorylation Deficiency 3
TTC8	Bardet-Biedl Syndrome 8
TTPA	Ataxia with Vitamin E Deficiency
TYMP	Myoneurogastrointestinal Encephalopathy Syndrome (MNGIE)
UBR1	Johanson-Blizzard Syndrome
USH1C	Usher Syndrome, Type 1C
USH2A	Usher Syndrome, Type 2A
VPS13A	Choreoacanthocytosis
VPS13B	Cohen Syndrome
VPS45	Congenital Neutropenia 5
VRK1	Pontocerebellar Hypoplasia Type 1A
VSX2	Microphthalmia, VSX2-Related
VWF	von Willebrand Disease, Types 2A, 2B, 2M, 2N and 3
WNT10A	Odontoonychodermal Dysplasia
XPA	Xeroderma Pigmentosum Group A
XPC	Xeroderma Pigmentosum Group C
ZFYVE26	Spastic Paraplegia 15

Table S4. GS Cases with Phenotypic Features Explained by Definitive Multiple Genetic Diagnoses

Study ID	HPO Terms	Diagnostic Finding Category	Gene Symbol / HGNC ID	Variants Identified	Genomic Position (hg19/GRCh37)	Zygosity	Parent of Origin	Variant Classification	Classification Criteria	Associated Disease / Inheritance
PKIG00155	HP:0004322 short stature; HP:0009803 short phalanx of the finger; HP:0000256 macrocephaly; HP:0002808 kyphosis; HP:0009826 limb undergrowth; HP:0003307 hyperlordosis; HP:0002373 febrile seizures	Diagnostic Finding	*COL2A1* / HGNC:2200	NM_001844.4:c.2257G>A p.(Gly753Ser)	NC_000012.11:g.48376329C>T	Hz	Assumed *De novo* (follow-up parental studies)	Pathogenic	PM6_Strong, PS4_Mod, PM1_Mod, PM2_Sup, PP3_Sup, PP4_Sup	*COL2A1* - related skeletal dysplasia / AD
		Diagnostic Finding	*PCDH19* / HGNC:14270	NM_001184880.1:c.2571_2572delGC p.?	NC_000023.10:g.99657566_99657567del	Hz	Paternal (follow-up parental studies)	Pathogenic	PVS1_VS, PS1_Strong, PM2_Sup	Developmental and epileptic encephalopathy 9 / XL
PKIG00180	HP:0001790 non-immune fetal hydrops; HP:0001999 abnormal facial shape; HP:0000369 low-set ears; HP:0000582 upslanted palpebral fissure; HP:0000969 edema; HP:0001541 ascites; HP:0002615 hypotension; HP:0000821 hypothyroidism; HP:0003112 abnormality of serum amino acid levels; HP:0004430 severe combined immunodeficiency; HP:0011682 perimembranous ventricular septal defect; HP:0000954 single transverse palmar crease; HP:0008905 rhizomelia; HP:0001561 polyhydramnios; HP:0001622 premature birth	Diagnostic Finding	n/a	seq[GRCh37] 21q11.2q22.3(14368147_48102246)x3 33.7 Mb dup of 21q11.2q22.3	NC_000021.8:g.14368147_48102246dup	n/a	Unknown	Pathogenic	Trisomy 21	Down syndrome / sporadic aneuploidy
		Diagnostic Finding	*GLA* / HGNC:4296	NM_000169.2:c.679C>T p.(Arg227Ter)	NC_000023.10:g.100653895G>A	Hz	Maternal (follow-up parental studies)	Pathogenic	PVS1_VS, PM2_Sup, PP4_Sup	Fabry disease; Fabry disease, cardiac variant / XL
PKIG00282[7]	HP:0001511 intrauterine growth restriction; HP:0001667 right ventricular hypertrophy; HP:0004969 peripheral pulmonary artery stenosis; HP:0001655 patent foramen ovale; HP:0005176 dysplastic aortic valve; HP:0012304 hypoplastic aortic arch; HP:0011675 arrhythmia; HP:0000822 hypertension; HP:0001943 hypoglycemia; HP:0002240 hepatomegaly; HP:0001999 abnormal facial shape; HP:0001939 abnormality of metabolism/homeostasis	Diagnostic Finding	n/a	seq[GRCh37] 16p13.11(15424126_16464025)x3 1.04 Mb dup of 16p13.11	NC_000016.9:g.15424126_16464025dup	n/a	Maternal	Likely Pathogenic	2A: 0.9 pts	16p13.11 microduplication syndrome / AD
		Diagnostic Finding	n/a	seq[GRCh37] 7q11.23(72650131_74146930)x1 1.5 Mb del of 7q11.23	NC000007.13:g.72650131_74146930del	Hz	*De novo*	Pathogenic	2A: 1 pt	Williams-Beuren syndrome / AD
		Other Finding Unrelated to Phenotype	*PRNP* / HGNC:9449	NM_000311.3:c.598G>A p.(Glu200Lys)	NC_000020.10:g.4680464G>A	Hz	Maternal	Pathogenic	PP1_Strong, PM1_Mod, PS3_Sup, PM2_Sup, PP3_Sup, PP4_Sup	Cerebral amyloid angiopathy, *PRNP* - related; Creutzfeldt-Jakob disease; Gerstmann-Straussler disease; Huntington disease-like 1; Fatal familial insomnia; Prion disease with protracted course / AD
PKIG00374[7]	HP:0000588 optic nerve colobomata; HP:0000193 bifid uvula; HP:0001250 seizures; HP:0007359 focal seizures; HP:0001488 bilateral ptosis; HP:0006934 congenital nystagmus; HP:0000545 myopia; HP:0000568 microphthalmia; HP:0000028 cryptorchidism; HP:0001263 global developmental delay; HP:0001344 absent speech; HP:0000750 delayed speech and language development; HP:0001249 intellectual disability; HP:0000618 blindness; HP:0000972 palmoplantar hyperkeratosis; HP:0000268 dolichocephaly; HP:0000276 long face; HP:0000369 low-set ears; HP:0001182 tapered finger; HP:0000953 hyperpigmentation of the skin; HP:0008619 bilateral sensorineural hearing impairment	Diagnostic Finding	*GJB2* / HGNC:4284	NM_004004.6:c.101T>C p.(Met34Thr)	NC_000013.10:g.20763620A>G	Hz	Unknown, confirmed *in trans* by IGV	Pathogenic	PM3_VS, PS4_Strong, PP1_Strong	*GJB2* - related deafness / AR
		Diagnostic Finding	*GJB2* / HGNC:4284	NM_004004.6:c.109G>A p.(Val37Ile)	NC_000013.10:g.20763612C>T	Hz		Pathogenic	PM3_VS, PS4_Strong, PP1_Strong	
		Diagnostic Finding	*PACS1* / HGNC:30032	NM_018026.4:c.607C>T p.(Arg203Trp)	NC_000011.9:g.65978677C>T	Hz	Unknown	Pathogenic	PS2_VS, PS3_Strong, PS4_Strong, PM2_Sup	Schuurs-Hoeijmakers syndrome / AD
PKIG00405	HP:0000969 dysplastic corpus callosum; HP:0001252 muscular hypotonia; HP:0000252 microcephaly; HP:0001270 motor delay; HP:0000750 delayed speech and language development; HP:0001249 intellectual disability; HP:0007018 attention deficit hyperactivity disorder; HP:0002910 elevated hepatic transaminases; HP:0000589 coloboma; HP:0000568 microphthalmia; HP:0000486 strabismus; HP:0000505 visual impairment; HP:0000668 hypodontia; HP:0001999 abnormal facial shape; HP:0000601 hypotelorism; HP:0000582 upslanted palpebral fissure; HP:0000368 low-set posterior rotated ears; HP:0000411 protruding ear; HP:0011800 hypoplasia of midface; HP:0012471 thick vermilion border; HP:0010803 everted upper lip vermilion; HP:0000331 short chin; HP:0001792 small nail; HP:0030084 clinodactyly; HP:0001845 overlapping toe; HP:0001531 failure to thrive in infancy; HP:0001511 intrauterine growth restriction; HP:0001562 oligohydramnios	Diagnostic Finding	*ABCA4* / HGNC:34	NM_000350.3:c.5882G>A p.(Gly1961Glu)	NC_000001.10:g.94473807C>T	Hm	Maternal & Paternal	Pathogenic with reduced penetrance	PM3_VS, PP1_Strong, PP3_Sup	Cone-rod dystrophy 3; Fundus flavimaculatus; Retinal dystrophy, early-onset severe; Retinitis pigmentosa 19; Stargardt disease 1 / AR
		Diagnostic Finding	*THOC6* / HGNC:28369	NM_024339.3:c.810+1G>A p.?	NC_000016.9:g.3077282G>A	Hm	Maternal & Paternal	Likely Pathogenic	PVS1_VS, PM2_Sup	Beaulieu-Boycott-Innes syndrome / AR
		Diagnostic Finding	*RP1* / HGNC:10263	NM_006269.1:c.5248G>T p.(Glu1750Ter)	NC_000008.10:g.55541690G>T	Hm	Maternal & Paternal	Likely Pathogenic	PVS1_VS, PM2_Sup	Retinitis pigmentosa 1 / AR
PKIG00596	HP:0001270 motor delay; HP:0000750 delayed speech and language development; HP:0001263 global developmental delay; HP:000082 hypertension; HP:0000482 microcornea; HP:0000508 ptosis; HP:0100324 sclerocornea; HP:0012751 abnormal basal ganglia MRI signal intensity; HP:0002153 hyperkalemia; HP:0001510 growth delay	Diagnostic Finding	*MMUT* / HGNC:7526	NM_000255.3:c.2200C>T p.(Gln734Ter)	NC_000006.11:g.49399494G>A	Hm	Unknown	Pathogenic	PVS1_Strong, PS4_Mod, PM3_Mod, PM2_Sup, PP1_Sup, PP4_Sup	Methylmalonic aciduria, mut(0) type / AR
		Diagnostic Finding	*KERA* / HGNC:6309	NM_007035.3:c.1026del p.?	NC_000012.11:g.91445157del	Hm	Unknown	Likely Pathogenic	PP1_ Strong, PM2_Sup, PM3_Sup	Cornea plana 2 / AR
PKIG01041	HP:0001263 global developmental delay; HP:0001319 congenital hypotonia; HP:0001269 hemiparesis	Diagnostic Finding	n/a	seq[GRCh37] 22q11.21(18843957_21544932)x3 1.5 Mb dup of 22q11.21	NC_000022.10:g.18843957_21544932dup	n/a	*De novo*	Pathogenic	2A: 1 pt	22q11.2 duplication syndrome / AD
		Diagnostic Finding	*CUL3* / HGNC:2553	NM_003590.5:c.264+1G>C p.?	NC_000002.11:g.225422375C>G	Hz	*De novo*	Likely Pathogenic	PVS1_VS, PM2_Sup	Neurodevelopmental disorder with or without autism or seizures; Pseudohypoaldosteronism, type IIE / AD
PKIG01194	HP:0001363 craniosynostosis; HP:0100324 scleroderma; HP:0000505 visual impairment; HP:0002828 multiple joint contracture; HP:0001159 syndactyly; HP:0001762 talipes equinovarus; HP:0001629 ventricular septal defect; HP:0005815 supranumerary ribs; HP:0008797 early ossification of capital femoral epiphyses; HP:0000774 narrow chest; HP:0012745 short palpebral fissure; HP:0007957 corneal opacity; HP:0000158 macroglossia; HP:0000356 abnormality of the outer ear; HP:0000347 micrognathia	Diagnostic Finding	n/a	seq[GRCh37] 16p13.11(15422726_16697685)x3 1.3 Mb dup of 16p13.11	NC_000016.9:g.15422726_16697685dup	n/a	Unknown	Likely Pathogenic	2A: 0.9 pts	16p13.11 microduplication syndrome / AD
		Diagnostic Finding	n/a	seq[GRCh37] 2q37.3(239521315_243067735)x1 3.5 Mb del of 2q37.3	NC_000002.11:g.239521315_243067735del	Hz	Unknown	Pathogenic	2A: 1 pt	2q37 deletion syndrome / AD
		Diagnostic Finding	n/a	seq[GRCh37] 9q33.2q34.3(123395115_141113114)x3 17.7 Mb dup of 9q33.2q34.3	NC_000009.11:g.123395115_141113114dup	n/a	Unknown	Pathogenic	3C: 0.9 pts, 4C: 0.3 pts; 5G: 0.1 pts	9q34 microduplication syndrome / AD
PKIG01276	HP:0001263 global developmental delay; HP:0001249 intellectual disability; HP:0012427 excessive femoral anteversion; HP:0001363 craniosynostosis; HP:0000297 facial hypotonia; HP:0005280 depressed nasal bridge; HP:0000431 wide nasal bridge; HP:0000506 telecanthus	Diagnostic Finding	n/a	seq[GRCh37] 15q11.2(22748146_23277545)x1 529.4 Kb del of 15q11.2	NC_000015.9:g.22748146_23277545del	Hz	Unknown	Pathogenic	2A: 1 pt	15q11.2 microdeletion syndrome / AD
		Diagnostic Finding	n/a	seq[GRCh37] 17p12(14089600_15486408)x3 1.4 Mb dup of 17p12	NC_000017.10:g.14089600_15486408dup	n/a	Unknown	Pathogenic	2A: 1 pt	Charcot-Marie-Tooth disease, type 1A / AD
		Diagnostic Finding	*POGZ* / HGNC:18801	NM_015100.4:c.2324_2325insTT p.?	NC_000001.10:g.151380627_151380628insAA	Hz	Maternal (follow-up parental studies)	Likely Pathogenic	PVS1_VS, PM2_Sup	White-Sutton syndrome / AD
PKIG01425	HP:0004322 short stature; HP:0001642 pulmonic stenosis; HP:0100633 esophagitis	Diagnostic Finding	*G6PC* / HGNC:4056	NM_000151.4:c.247C>T p.(Arg83Cys)	NC_000017.10:g.41055964C>T	Hm	Maternal & Paternal	Pathogenic	PS3_Strong, PM1_Mod, PM3_Mod, PP3_Sup, PP4_Sup	Glycogen storage disease Ia / AR
		Diagnostic Finding	*PTPN11* / HGNC:9644	NM_002834.4:c.1492C>T p.(Arg498Trp)	NC_000012.11:g.112926872C>T	Hz	*De novo*	Pathogenic	PS2_VS, PS3_Strong, PM5_Mod, PM2_Sup, PP3_Sup	LEOPARD syndrome 1; Metachondromatosis; Noonan syndrome 1 / AD
PKIG01849	HP:0000252 microcephaly; HP:0001276 hypertonia; HP:0001510 growth delay; HP:0001511 intrauterine growth restriction; HP:0001531 failure to thrive in infancy; HP:0007068 inferior vermis hypoplasia; HP:0000028 cryptorchidism; HP:0000368 low-set posteriorly rotated ears; HP:0008872 feeding difficulties in infancy; HP:0002020 gastroesophageal reflux; HP:0002616 aortic root dilation	Diagnostic Finding	*KAT6A* / HGNC:13013	NM_006766.5:c.3385C>T p.(Arg1129Ter)	NC_000008.10:g.41792353G>A	Hz	*De novo*	Pathogenic	PVS1_VS, PS2_VS, PM2_Sup, PP4_Sup	Arboleda-Tham syndrome / AD
		Diagnostic Finding	*PAH* / HGNC:8582	NM_000277.3:c.527G>T p.(Arg176Leu)	NC_000012.11:g.103249093C>A	Hz	Paternal	Pathogenic	PM3_VS, PS3_Strong, PP4_Mod, PM2_Sup	Phenylketonuria / AR
		Diagnostic Finding	*PAH* / HGNC:8582	NM_000277.3:c.898G>T p.(Ala300Ser)	NC_000012.11:g.103245479C>A	Hz	Maternal	Pathogenic	PM3_VS, PS3_Strong, PP3_Sup, PP4_Sup	
PKIG02092	HP:0001531 failure to thrive in infancy; HP:0001270 motor delay; HP:0000750 delayed speech and language development; HP:0001263 global developmental delay; HP:0001319 neonatal hypotonia; HP:0006934 congenital nystagmus; HP:0000565 esotropia; HP:0000047 hypospadias; HP:0000028 cryptorchidism; HP:0008872 feeding difficulties in infancy; HP:0002015 dysphagia	Diagnostic Finding	n/a	seq[GRCh37] 20q13.2q13.33(51050514_62960210)x3 11.9 Mb dup of 20q13.2q13.33	NC_000020.10:g.51050514_62960210dup	n/a	Unknown	Pathogenic	3C: 0.9 pts, 5G: 0.1 pts	Partial trisomy of the distal region of chromosome 20q / AD Sporadic
		Diagnostic Finding	n/a	seq[GRCh37] 13q11q12.3(19005309_30888810)x3 11.9 Mb dup of 13q11q12.3	NC_000013.10:g.19005309_30888810dup	n/a	Unknown	Pathogenic	3C: 0.9 pts, 5G: 0.1 pts	Proximal trisomy 13q / AD Sporadic
PKIG02101	HP:0011675 arrhythmia; HP:0002878 respiratory failure; HP:0004902 congenital lactic acidosis; HP:0001250 seizures; HP:0002104 apnea; HP:0006579 prolonged neonatal jaundice; HP:0002153 hyperkalemia; HP:0002917 hypomagnesemia; HP:0001987 hyperammonemia; HP:0001760 abnormality of the foot; HP:0100519 anuria; HP:0100520 oliguria; HP:0012211 abnormal renal physiology; HP:0002615 hypotension	Diagnostic Finding	*RORB* / HGNC:10259	NM_006914.4:c.286G>T p.(Glu96Ter)	NC_000009.11:g.77257380G>T	Hz	Unknown	Likely Pathogenic	PVS1_VS, PM2_Sup	Susceptibility to idiopathic generalized epilepsy 15 / AD
		Diagnostic Finding	n/a	seq[GRCh37] Xp11.4(38079631_38680130)x0 600.5 Kb del of Xp11.4	NC_000023.10:g.38079631_38680130del	Hemi	Unknown	Pathogenic	2A: 1 pt	Ornithine transcarbamylase deficiency / XL
PKIG00063	HP:0011169 generalized clonic seizures; HP:0001562 oligohydramnios; HP:0001511 intrauterine growth restriction; HP:0000568 microphthalmia; HP:0000519 congenital cataract; HP:0000035 abnormality of the testis; HP:0011625 multiple muscular ventricular septal defects; HP:0011621 Gerbode ventricular septal defect; HP:0005483 abnormality of the epiglottis; HP:0001601 laryngomalacia; HP:0002779 tracheomalacia; HP:0001508 failure to thrive; HP:0002098 respiratory distress; HP:0002099 asthma; HP:0000846 adrenal insufficiency; HP:0000821 hypothyroidism; HP:0008213 gonadotropin deficiency; HP:0030260 microphallus; HP:0010535 sleep apnea; HP:0000407 sensorineural hearing impairment; HP:0001953 diabetic ketoacidosis; HP:0001269 hemiparesis; HP:0000100 nephrotic syndrome; HP:0001263 global developmental delay; HP:0001252 hypotonia; HP:0003074 hyperglycemia	Diagnostic Finding	n/a	seq[GRCh37] 21q11.2q22.3(15348044_48102578)x3 32.8 Mb dup of 21q11.2q22.3	NC_000021.8:g.15348044_48102578dup	n/a	Unknown	Pathogenic	Trisomy 21	Down syndrome / Aneuploidy
		Diagnostic Finding	n/a	seq[GRCh37] 2q11.1q12.1(95346304_104100003)x3 8.8 Mb dup of 2q11.1q12.1	NC_000002.11:g.95346304_104100003dup	n/a	Unknown	Likely Pathogenic	3C: 0.9	2q11.1q12.1 duplication / AD
		Diagnostic Finding	*ERCC8* / HGNC:3439	NM_000082.3:c.613G>C p.(Ala205Pro)	NC_000005.9:g.60198273C>G	Hm	Unknown	Likely Pathogenic	PM3_Strong, PP1_Mod, PS3_Sup	Cockayne syndrome, type A; UV-sensitive syndrome 2 / AR
PKIG00099	HP:0000729 autistic behavior; HP:0007018 attention deficit hyperactivity disorder; HP:0012447 abnormal myelination; HP:0007305 CNS demyelination; HP:0001251 ataxia; HP:0000505 visual impairment; HP:0200026 ocular pain; HP:0000649 abnormality of visual evoked potentials; HP:0008619 bilateral sensorineural hearing impairment; HP:0001265 hyporeflexia; HP:0002019 constipation	Diagnostic Finding	n/a	XYY	n/a	n/a	*De novo*	Pathogenic	Y Disomy	Jacobs syndrome / Sporadic Aneuploidy
		Diagnostic Finding	*ACOX1* / HGNC:119	NM_004035.6:c.710A>G p.(Asn237Ser)	NC_000017.10:g.73951711T>C	Hz	*De novo*	Likely Pathogenic	PS2_Strong, PS3_Sup, PM2_Sup, PP3_Sup	Mitchell syndrome / AD

Abbreviations: HGNC, HUGO Gene Nomenclature Committee; Hz, Heterozygous; Hm, Homozygous; Hp, Heteroplasmic; Hemi, Hemizygous; IGV, Integrative Genomics Viewer; VS, very strong; Mod, moderate; Sup, supporting; AD, autosomal dominant; AR, autosomal recessive; XL, X-linked.

Table S5. GS Cases with Phenotypic Features Explained by Presumed Multiple Genetic Diagnoses

Study ID	HPO Terms	Diagnostic Finding Category	Gene Symbol / HGNC ID	Variants Identified	Genomic Position (hg19/GRCh37)	Zygosity	Parent of Origin	Variant Classification	Classification Criteria	Associated Disease / Inheritance
PKIG00165[7]	HP:0001510 growth delay; HP:0011344 severe global developmental delay; HP:0001531 failure to thrive in infancy; HP:0008872 feeding difficulties in infancy; HP:0001274 agenesis of corpus callosum; HP:0001250 seizures; HP:0000822 hypertension; HP:0005957 breathing dysregulation; HP:0003128 lactic acidosis; HP:0002878 respiratory failure; HP:0012416 hypercapnia; HP:0002098 respiratory distress; HP:0002093 respiratory insufficiency	Diagnostic Finding	*KCNH2* / HGNC:6251	NM_000238.3:c.774_789del p.?	NC_000007.13:g.150655274_150655289del	Hz	Paternal	Pathogenic	PVS1_VS, PS1_Mod, PM2_Sup	Long QT syndrome 2; Short QT syndrome 1 / AD
		Diagnostic Finding	*SLC25A19* / HGNC:14409	NM_021734.4:c.115A>T p.(Ile39Phe)	NC_000017.10:g.73282731T>A	Hz	Paternal	Uncertain Significance	PM2_Sup, PP3_Sup	Microcephaly, Amish type; Thiamine metabolism dysfunction syndrome 4 / AR
		Diagnostic Finding	*SLC25A19* / HGNC:14409	NM_021734.4:c.433C>A p.(Arg145Ser)	NC_000017.10:g.73279530G>T	Hz	Maternal	Uncertain Significance	PM2_Sup, PP3_Sup	
PKIG00217	HP:0000410 mixed hearing impairment; HP:0004322 short stature; HP:0000540 hypermetropia; HP:0009237 short 5th finger; HP:0004209 clinodactyly of the 5th finger; HP:0001609 hoarse voice	Diagnostic Finding	*TNC* / HGNC:5318	NM_002160.3:c.5692G>A p.(Val1898Ile)	NC_000009.11:g.117797578C>T	Hz	Maternal (follow-up parental studies)	Uncertain Significance	PM2_Sup	Deafness 56 / AD
		Diagnostic Finding	*ANKRD11* / HGNC:21316	NM_013275.5:c.2288_2289del p.?	NC_000016.9:g.89350661_89350662del	Hz	Assumed *de novo* (follow-up parental studies)	Pathogenic	PVS1_VS, PS4_Sup, PM2_Sup	KBG syndrome / AD
		Other Finding Unrelated to Phenotype	*HFE* / HGNC:4886	NM_000410.3:c.187C>G p.(His63Asp)	NC_000006.11:g.26091179C>G	Hm	Unknown	Pathogenic	PM3_VS, PS3_Mod, PP4_Sup	Hemochromatosis / AR
PKIG00252	HP:0030300 small pituitary gland; HP:0001270 motor delay; HP:0000750 delayed speech and language development; HP:0001344 absent speech; HP:0002187 profound intellectual disability; HP:0002072 chorea; HP:0008947 infantile muscular hypotonia; HP:0007359 focal seizure; HP:0007335 recurrent encephalopathy; HP:0000565 esotropia; HP:0000023 inguinal hernia; HP:0100716 self-injurious behavior	Diagnostic Finding	*CACNA1A* / HGNC:1388	NM_001127221.1:c.2042_2043del p.?	NC_000019.9:g.13414645_13414646del	Hz	*De novo*	Pathogenic	PVS1_VS, PS2_Mod, PP1_Mod, PM2_Sup	Early infantile epileptic encephalopathy 42; Episodic ataxia, type 2; Familial hemiplegic migraine 1; Spinocerebellar ataxia 6 / AD
		Diagnostic Finding	*GABBR2* / HGNC:4507	NM_005458.7:c.1724C>A p.(Thr575Asn)	NC_000009.11:g.101133792G>T	Hz	*De novo*	Uncertain Significance	PM2_Sup, PP3_Sup	Early infantile epileptic encephalopathy 59; Neurodevelopmental disorder with poor language and loss of hand skills / AD
PKIG00301	HP:0001270 motor delay; HP:0000750 delayed speech and language development; HP:0001249 intellectual disability; HP:0001251 ataxia; HP:0008947 infantile muscular hypotonia; HP:0000252 microcephaly; HP:0000643 blepharospasm; HP:0000508 ptosis; HP:0000505 visual impairment; HP:0008572 external ear malformation; HP:0001162 postaxial hand polydactyly; HP:0002019 constipation; HP:0001531 failure to thrive in infancy; HP:0004322 short stature; HP:0001999 abnormal facial shape; HP:0000464 abnormality of the neck; HP:0002381 aphasia	Diagnostic Finding	n/a	seq[GRCh37] 3p26.1p25.3(8300214_10189085)x1 1.9 Mb del of 3p26.1p25.3	NC_000003.11:g.8300214_10189085del	Hz	*De novo*	Pathogenic	2A: 1 pt	3p- syndrome / AD
		Diagnostic Finding	*GRIN2B* / HGNC:4586	NM_000834.3:c.1427A>G p.(Tyr476Cys)	NC_000012.11:g.13768500T>C	Hz	*De novo*	Uncertain Significance	PM2_Sup, PP3_Sup	Developmental and epileptic encephalopathy 27; Intellectual developmental disorder 6 with or without seizures / AD
PKIG00439	HP:0002721 immunodeficiency; HP:0100753 schizophrenia; HP:0001332 dystonia; HP:0002401 stroke-like episodes; HP:0007018 attention deficit hyperactivity disorder; HP:0001263 global developmental delay; HP:0002376 developmental regression; HP:0001249 intellectual disability; HP:0001251 ataxia; HP:0001252 muscular hypotonia; HP:0001298 encephalopathy; HP:0002315 headache; HP:0002076 migraine; HP:0040081 abnormal levels of creatine kinase in the blood; HP:0003348 hyperalaninemia; HP:0003542 increased serum pyruvate; HP:0001946 ketosis; HP:0000505 visual impairment; HP:0000924 abnormality of the skeletal system; HP:0000925 abnormality of the vertebral column; HP:0001382 joint hypermobility; HP:0002650 scoliosis; HP:0001597 abnormality of the nail; HP:0002019 constipation; HP:0001508 failure to thrive; HP:0002013 vomiting; HP:0001561 polyhydramnios	Diagnostic Finding	*KDM6B* / HGNC:29012	NM_001080424.1:c.1069G>A p.(Gly357Arg)	NC_000017.10:g.7750582G>A	Hz	Maternal	Uncertain Significance	none	Neurodevelopmental disorder with coarse facies and mild distal skeletal abnormalities / AD
		Diagnostic Finding	*GATA2* / HGNC:4171	NM_032638.4:c.1017+572C>T p.?	NC_000003.11:g.128202131G>A	Hz	Unknown	Pathogenic	PP1_Strong, PS4_Mod, PM1_Mod, PS3_Sup, PP4_Sup	Emberger syndrome; Immunodeficiency 21; Susceptibility to myelodysplastic syndrome and acute myeloid leukemia / AD
PKIG00483	HP:0001249 intellectual disability; HP:0002363 abnormality of brainstem morphology; HP:0002814 abnormality of the lower limb; HP:0001155 abnormal of the hand; HP:0002465 poor speech	Diagnostic Finding	*DYM* / HGNC:21317	seq[GRCh37] 18q21.1(46714849_46903615)x1 188.8 Kb intragenic del of NM_017653(*DYM*) exons 6-13	NC_000018.9:g.46714849_46903615del	Hz	Unknown	Pathogenic	Heterozygous intragenic out-of-frame deletion in an autosomal recessive gene	Dyggve-Melchior-Clausen disease; Smith-McCort dysplasia / AR
		Diagnostic Finding	*DYM* / HGNC:21317	seq[GRCh37] 18q21.1(46778800_46783874)x0 5.1 Kb intragenic del of NM_017653(*DYM*) exon 13	NC_000018.9:g.46778800_46783874del	Hm	Unknown	Pathogenic	Homozygous intragenic out-of-frame deletion in an autosomal recessive gene	
		Diagnostic Finding	*PTCHD1* / HGNC:26392	NM_173495.3:c.1636C>G p.(Gln546Glu)	NC_000023.10:g.23411271C>G	Hemi	Unknown	Uncertain Significance	PM2_Sup	Susceptibility to autism 4 / XLR
PKIG01019	HP:0010945 fetal pyelectasis; HP:0000519 congenital cataract; HP:0000589 coloboma; HP:0000568 microphthalmia; HP:0000505 visual impairment; HP:0002705 high narrow palate; HP:0003265 hyperbilirubinemia; HP:0000952 jaundice; HP:0000126 hydronephrosis; HP:0012210 abnormal renal morphology; HP:0007598 bilateral single transverse palmar creases; HP:0002093 respiratory insufficiency; HP:0006704 abnormality of the coronary arteries	Diagnostic Finding	n/a	seq[GRCh37] 14q31.2q32.13(84142520_95801685)x1 11.7 Mb del of 14q31.2q32.13	NC_000014.8:g.84142520_95801685del	Hz	Assumed *de novo* (follow-up parental studies)	Pathogenic	2A: 1 pt	14q31.214q deletion from 14q31 to 14q32.1 / AD
		Diagnostic Finding	*RAB23* / HGNC:14263	NM_183227.2:c.-66+4614dup p.?	NC_000006.11:g.57081512dup	Hz	Unknown	Uncertain Significance	PM2_Sup	Carpenter syndrome / AR
		Diagnostic Finding	*RAB23* / HGNC:14263	NM_183227.2:c.434T>A p.(Leu145Ter)	NC_000006.11:g.57059615A>T	Hz	Unknown	Pathogenic	PVS1_VS, PM3_Strong, PP1_Sup, PP4_Sup	
PKIG01287[7]	HP:0001251 ataxia; HP:0001250 seizure; HP:0001298 encephalopathy; HP:0000316 hypertelorism; HP:0005280 depressed nasal bridge; HP:0000455 broad nasal tip	Diagnostic Finding	n/a	seq[GRCh37] 16p13.11(14868694_16366925)x3 1.5 Mb dup of 16p13.11	NC_000016.9:g.14868694_16366925dup	n/a	Maternal	Likely Pathogenic	2A: 0.9 pts	16p13.11 microduplication syndrome / AD
		Diagnostic Finding	*PC* / HGNC:8636	NM_000920.4:c.2000A>G p.(Lys667Arg)	NC_000011.9:g.66618734T>C	Hm	Maternal & Paternal	Uncertain Significance	PM2_Sup	Pyruvate carboxylase deficiency / AR
PKIG01703	HP:0001531 failure to thrive in infancy; HP:0008947 infantile muscular hypotonia; HP:0001263 global developmental delay; HP:0002019 constipation; HP:0000243 trigonocephaly; bitemporal narrowing; HP:0003196 short nose; anteverted nares; smooth philtrum; HP:0000369 low-set ears; HP:0001382 joint hypermobility	Diagnostic Finding	*PPM1D* / HGNC:9277	NM_003620.4:c.1224dup p.?	NC_000017.10:g.58734166dup	Hz	Unknown	Likely Pathogenic	PVS1_VS, PM2_Sup	Jansen de Vries syndrome / AD
		Diagnostic Finding	*SCAPER* / HGNC:13081	NM_020843.4:c.1382A>T p.(Asp461Val)	NC_000015.9:g.77059296T>A	Hz	Unknown	Uncertain Significance	PM2_Sup	Intellectual developmental disorder and retinitis pigmentosa / AR
		Diagnostic Finding	*SCAPER* / HGNC:13081	NM_020843.4:c.1250C>T p.(Ser417Phe)	NC_000015.9:g.77059428G>A	Hz	Maternal	Uncertain Significance	PM2_Sup	
PKIG01847	HP:0000201 Pierre Robin sequence; HP:0001257 spasticity; HP:0001264 spastic diplegia; HP:0100021 cerebral palsy; HP:0001263 global developmental delay; HP:0005684 distal arthrogryposis; HP:0000474 thickened nuchal skin fold; HP:0007598 bilateral single transverse palmar creases; HP:0011913 lumbar hypertrichosis; HP:0002144 tethered cord; HP:0007359 focal seizures; HP:0000220 velopharyngeal insufficiency; HP:0040083 toe walking; HP:0003326 myalgia; HP:0100716 self-injurious behavior; HP:0002360 sleep disturbance; HP:0004322 short stature; HP:0007018 attention deficit hyperactivity disorder	Diagnostic Finding	*PIEZO2* / HGNC:26270	seq[GRCh37] 18p11.21(10911731_10923591)x1 11.9 Kb intragenic del of NM_022068(*PIEZO2*) intron 3	NC_000018.9:g.10911731_10923591del	Hz	Unknown	Uncertain Significance	4E: 0.1 pts	Distal arthrogryposis type 3; Distal arthrogryposis type 5; Distal arthrogryposis with impaired proprioception and touch / AD; AR
		Diagnostic Finding	*WAC* / HGNC:17327	NM_016628.5:c.265_266del p.?	NC_000010.10:g.28824671_28824672del	Hz	Unknown	Pathogenic	PVS1_VS, PS2_Sup, PM2_Sup	Desanto-Shinawi syndrome / AD
PKIG01887[7]	HP:0001263 global developmental delay; HP:0001270 motor delay; HP:0000750 delayed speech and language development; HP:0002376 developmental regression; HP:0001249 intellectual disability; HP:0001252 muscular hypotonia; HP:0001257 spasticity; epileptic activity; HP:0000252 microcephaly; HP:0005216 chewing difficulties; HP:0002015 dysphagia; HP:0002033 poor suck; HP:0002019 constipation; HP:0002020 gastroesophageal reflux; HP:0000708 behavioral abnormality; HP:0003763 bruxism; HP:0012169 self-biting; HP:0000733 abnormal repetitive mannerisms; HP:0000540 hypermetropia; HP:0010296 ankyloglossia	Diagnostic Finding	*MECP2* / HGNC:6990	seq[GRCh37] Xq28(153272031_153296030)x1 24.0 Kb intragenic del of NM_004992.3(*MECP2*) exon 3	NC_000023.10:g.153272031_153296030del	Hz	Unknown	Pathogenic	2D-2: 0.9pts, 5G: 0.1 pts	Neonatal severe encephalopathy; Syndromic intellectual developmental disorder 13; Syndromic intellectual developmental disorder, Lubs type; Rett syndrome; Atypical Rett syndrome; Rett syndrome, preserved speech variant / XLD; XLR
		Diagnostic Finding	*DNM1* / HGNC:2972	NM_004408.4:c.352G>C p.(Val118Leu)	NC_000009.11:g.130980977G>C	Hz	Unknown	Uncertain Significance	PM2_Sup	Developmental and epileptic encephalopathy 31A; Developmental and epileptic encephalopathy 31B / AD; AR
PKIG02037	HP:0007359 focal seizures; HP:0002349 focal seizures without impairment of consciousness or awareness; HP:0000729 autistic behavior; HP:0007165 periventricular gray matter heterotopia; HP:0001344 absent speech; HP:0001249 intellectual disability; HP:0001263 global developmental delay; HP:0001093 optic nerve dysplasia; HP:0100704 cortical visual impairment; HP:0000011 neurogenic bladder; HP:0000175 cleft palate; HP:0001319 neonatal hypotonia; HP:0000405 conductive hearing impairment; HP:0002719 recurrent infections; HP:0000733 stereotypic behavior; HP:0002144 tethered cord; HP:0040083 toe walking	Diagnostic Finding	*MT-TL1* / HGNC:7490	NC_012920.1:m.3243A>G	NC_012920.1:m.3243A>G	Hp	*De novo*	Pathogenic	PS3_Strong, PS4_Strong, PP1_Mod, PP3_Sup	Mitochondrial encephalomyopathy, lactic acidosis and stroke-like episodes (MELAS) / Mitochondrial
		Diagnostic Finding	*DMXL2* / HGNC:2938	NM_001174116.2:c.7268G>T p.(Arg2423Ile)	NC_000015.9:g.51763544C>A	Hz	Maternal	Uncertain Significance	PM2_Sup	Developmental and epileptic encephalopathy 81 / AR
		Diagnostic Finding	*DMXL2* / HGNC:2938	NM_001174116.2:c.5974G>A p.(Asp1992Asn)	NC_000015.9:g.51773329C>T	Hz	Paternal	Uncertain Significance	BP4_Sup	
PKIG02059	HP:0001263 global developmental delay; HP:0002376 developmental regression; HP:0002194 delayed gross motor development; HP:0001290 hypotonia; HP:0001875 neutropenia; HP:0002015 dysphagia; HP:0004755 supraventricular tachycardia; HP:0000821 hypothyroidism; HP:0002189 excessive daytime sleepiness	Diagnostic Finding	*COQ8A* / HGNC:16812	NM_020247.5:c.1651G>A p.(Glu551Lys)	NC_000001.10:g.227173033G>A	Hz	Maternal	Likely Pathogenic	PM3_Strong, PM2_Sup, PP4_Sup	Primary coenzyme Q10 deficiency 4 / AR
		Diagnostic Finding	*COQ8A* / HGNC:16812	NM_020247.5:c.901C>T p.(Arg301Trp)	NC_000001.10:g.227170426C>T	Hz	Unknown	Likely Pathogenic	PM3_Strong, PM2_Sup, PP4_Sup	
		Diagnostic Finding	*CIC* / HGNC:14214	NM_015125.4:c.298G>A p.(Ala100Thr)	NC_000019.9:g.42791238G>A	Hz	Unknown	Uncertain Significance	PM2_Sup, BP4_Sup	Intellectual developmental disorder 45 / AD

Abbreviations: HGNC, HUGO Gene Nomenclature Committee; Hz, Heterozygous; Hm, Homozygous; Hp, Heteroplasmic; Hemi, Hemizygous; VS, very strong; Mod, moderate; Sup, supporting; AD, autosomal dominant; AR, autosomal recessive; XLD, X-linked dominant; XLR, X-linked recessive.

Table S6. GS Cases with a Definitive Genetic Diagnosis and a Reportable Secondary Finding

Study ID	HPO Terms	Diagnostic Finding Category	Gene Symbol / HGNC ID	Variants Identified	Genomic Position (hg19/GRCh37)	Zygosity	Parent of Origin	Variant Classification	Classification Criteria	Associated Disease / Inheritance
PKIG00531	HP:0001263 global developmental delay; HP:0100021 cerebral palsy; HP:0008936 muscular hypotonia of the trunk; HP:0010535 sleep apnea; HP:0000175 cleft palate; HP:0000347 micrognathia; HP:0008872 feeding difficulties in infancy; HP:0000565 esotropia; HP:0001999 abnormal facial shape	Diagnostic Finding	*SATB2* / HGNC:21637	NM_015265.3:c.1705dup p.?	NC_000002.11:g.200173518dup	Hz	*De novo*	Pathogenic	PVS1_Strong, PS2_Strong, PM2_Sup	Glass syndrome / AD
		Other Finding Unrelated to Phenotype	*PALB2* / HGNC:26144	NM_024675.4:c.2167_2168del p.?	NC_000016.9:g.23641307_23641308del	Hz	Maternal	Pathogenic	PVS1_VS, PS4_Strong, PM5_Sup	Susceptibility to pancreatic cancer and familial breast-ovarian cancer / AD
PKIG00779	HP:0001251 ataxia; HP:0001324 muscle weakness; HP:0001000 abnormality of skin pigmentation; HP:0008064 ichthyosis	Diagnostic Finding	n/a	seq[GRCh37] Xp22.31(6450730_8137629)x0 1.6 Mb del of X22.31	NC_000023.9:g.6450730_813762del	Hemi	Unknown	Pathogenic	2A: 1 pt	Ichthyosis / XL
		Other Finding Unrelated to Phenotype	*HFE* / HGNC:4886	NM_000410.3:c.187C>G p.(His63Asp)	NC_000006.11:g.26091179C>G	Hm	Unknown	Pathogenic, reduced penetrance	PM3_VS, PS3_Mod, PP4_Sup	Hemochromatosis, type 1 / AR
PKIG00800	HP:0007018 attention deficit disorder; HP:0002194 delayed gross motor development; HP:0011451 congenital microcephaly; HP:0100543 cognitive impairment; HP:0001999 abnormal facial shape; HP:0001263 global developmental delay	Diagnostic Finding	*VPS13B* / HGNC:2183	seq[GRCh37] 8q22.2 (100076524_100109110)x0 32.6 Kb intragenic del of NM_017890(*VPS13B*) exon 4	NC_000008.10:g.100076524_100109110del	Hm	Unknown	Likely Pathogenic	Homozygous intragenic out-of-frame deletion in autosomal recessive gene	Cohen syndrome / AR
		Other Finding Unrelated to Phenotype	*COL10A1* / HGNC:2185	NM_000493.3:c.1616_1617del p.?	NC_000006.11:g.116441662_116441663del	Hz	Unknown	Likely Pathogenic	PVS1_VS, PM2_Sup	Metaphyseal chondrodysplasia, Schmid type / AD
PKIG01089	HP:0001257 spasticity; HP:0000924 abnormality of the skeletal system; HP:0001382 joint hypermobility; HP:0002828 multiple joint contractures; HP:0002650 scoliosis; HP:0002659 increased susceptibility to fractures; HP:0001622 premature birth; HP:0001623 breech presentation; HP:0006528 chronic lung disease; HP:0000952 jaundice; HP:0002194 delayed gross motor development; HP:0001290 generalized hypotonia; HP:0001537 umbilical hernia; HP:0000975 hyperhidrosis; HP:0003401 paresthesia	Diagnostic Finding	*MT-TL1* / HGNC:7490	NC_012920.1:m.3243A>G	NC_012920.1:m.3243A>G	Hp	Maternal	Pathogenic	PS3_Strong, PS4_Strong, PP1_Mod, PP3_Sup	Mitochondrial encephalomyopathy, lactic acidosis and stroke-like episodes (MELAS) / Mitochondrial
		Other Finding Unrelated to Phenotype	*HFE* / HGNC:4886	NM_000410.3:c.187C>G p.(His63Asp)	NC_000006.11:g.26091179C>G	Hz	Paternal	Pathogenic, reduced penetrance	PM3_VS, PS3_Mod, PP4_Sup	Hemochromatosis, type 1 / AR
		Other Finding Unrelated to Phenotype	*HFE* / HGNC:4886	NM_000410.3:c.845G>A p.(Cys282Tyr)	NC_000006.11:g.26093141G>A	Hz	Maternal	Pathogenic, reduced penetrance	PS3_Strong, PS4_Strong, PM3_Strong, PP4_Sup	
PKIG01298	HP:0001263 global developmental delay; HP:0001250 seizures; HP:0007359 focal epilepsy; HP:0011451 microcephaly; HP:0100702 arachnoid cyst; HP:0008947 infantile muscular hypotonia; HP:0004322 short stature	Diagnostic Finding	*ARF1* / HGNC:652	NM_001024226.1:c.296G>A p.(Arg99His)	NC_000001.10:g.228285328G>A	Hz	*De novo*	Likely Pathogenic	PS2_Strong, PM2_Sup, PP3_Sup	Periventricular nodular heterotopia 8 / AD
		Other Finding Unrelated to Phenotype	*COL4A4* / HGNC:2206	NM_000092.5:c.3452G>C p.(Gly1151Ala)	NC_000002.11:g.227906917C>G	Hz	Maternal	Likely Pathogenic	PM1_Mod, PM3_Mod, PM5_Sup, PP3_Sup	Alport syndrome 2; Hematuria, familial benign / AD; AR
PKIG01458	HP:0001511 intrauterine growth restriction; HP:0001999 abnormal facial shape; HP:0001518 small for gestational age; HP:0001531 failure to thrive in infancy; HP:0010296 ankyloglossia; HP:0000960 sacral dimple; HP:0001537 umbilical hernia; HP:0000347 micrognathia; HP:0000319 smooth philtrum; HP:0000953 hyperpigmentation of the skin; HP:0001622 preterm birth	Diagnostic Finding	n/a	seq[GRCh37] 5q35.1q35.3(172068830_180915260)x3 8.8 Mb dup of 5q35.1q35.3	NC_000005.9:g.172068830_180915260dup	n/a	Unknown	Pathogenic	2A: 1 pt	5q35 microduplication syndrome / AD
		Other Finding Unrelated to Phenotype	*MT-TL1* / HGNC:7490	NC_012920.1:m.3243A>G	NC_012920.1:m.3243A>G	Hp	Unknown	Pathogenic	PS3_Strong, PS4_Strong, PP1_Mod, PP3_Sup	Mitochondrial encephalomyopathy, lactic acidosis and stroke-like episodes (MELAS) / Mitochondrial
PKIG01570	HP:0000717 autism; HP:0001943 hypoglycemia; HP:0002240 hepatomegaly; HP:0002149 hyperuricemia; HP:0003077 hyperlipidemia; HP:0001875 neutropenia; HP:0001263 global developmental delay	Diagnostic Finding	*SLC37A4* / HGNC:4061	NM_001164277.1:c.1015G>T p.(Gly339Cys)	NC_000011.9:g.118896009C>A	Hz	Maternal	Pathogenic	PS3_Strong, PM1_Mod, PM5_Mod, PP4_Sup, PP1_Sup	Congenital disorder of glycosylation, type IIw; Glycogen storage disease Ib; Glycogen storage disease Ic / AD; AR
		Diagnostic Finding	*SLC37A4* / HGNC:4061	NM_001164277.1:c.1042_1043del p.?	NC_000011.9:g.118895981_118895982del	Hz	Paternal	Pathogenic	PVS1_VS, PM3_VS, PP4_Sup	
		Other Finding Unrelated to Phenotype	*TREX1* / HGNC:12269	NM_033629.6:c.341G>A p.(Arg114His)	NC_000003.11:g.48508395G>A	Hz	Maternal	Pathogenic	PS3_Strong, PM3_Mod, PM5_Mod, PP3_Sup, PP4_Sup	Aicardi-Goutieres syndrome 1; Chilblain lupus; Retinal vasculopathy with cerebral leukoencephalopathy and systemic manifestations / AD; AR
PKIG01739	HP:0002652 skeletal dysplasia; HP:0001591 bell-shaped thorax; HP:0003026 short long bone; HP:0006487 bowing of the long bones; HP:0003084 fractures of the long bones; HP:0002827 hip dislocation; HP:0100711 abnormality of the thoracic spine; HP:0000347 micrognathia; HP:0001631 atrial septal defect; HP:0002092 pulmonary hypertension; HP:0002089 pulmonary hypoplasia; HP:0001643 patent ductus arteriosus; HP:0001659 aortic regurgitation	Diagnostic Finding	*SOX9* / HGNC:11204	NM_000346.4:c.829G>T p.(Glu277Ter)	NC_000017.10:g.70119827G>T	Hz	Unknown	Likely Pathogenic	PVS1_VS, PM2_Sup	Acampomelic campomelic dysplasia; Campomelic dysplasia; Campomelic dysplasia with autosomal sex reversal / AD
		Other Finding Unrelated to Phenotype	*GLMN* / HGNC:14373	NM_053274.3:c.1323dup p.?	NC_000001.10:g.92729268dup	Hz	Unknown	Likely Pathogenic	PVS1_VS	Glomuvenous malformations / AD
PKIG02010	HP:0012469 infantile spasms; HP:0200134 epileptic encephalopathy; HP:0002197 generalized seizure; HP:0001249 intellectual disability; HP:0002376 developmental regression; HP:0001257 spasticity HP:0100021 cerebral palsy; HP:0011445 athetoid cerebral palsy; HP:0002072 chorea; HP:0001290 generalized hypotonia; HP:0000483 astigmatism; HP:0000098 tall stature; HP:0001929 reduced factor XI activity; HP:0000325 triangular face; HP:0002705 high, arched palate; HP:0003186 inverted nipples; HP:0002164 nail dysplasia; HP:0011300 broad fingertips	Diagnostic Finding	*F11* / HGNC:3529	NM_000128.4:c.1275_1281dup p.?	NC_000004.11:g.187205385_187205391dup	Hz	Maternal	Pathogenic	PVS1_VS, PM3_Mod, PM2_Sup	Factor XI deficiency / AD; AR
		Diagnostic Finding	*F11* / HGNC:3529	NM_000128.4:c.166T>C p.(Cys56Arg)	NC_000004.11:g.187192873T>C	Hz	Paternal	Pathogenic	PS3_Strong, PM3_Mod, PM5_Mod, PM2_Sup, PP3_Sup, PP4_Sup	
		Other Finding Unrelated to Phenotype	*DDX41* / HGNC:18674	NM_016222.4:c.1098+2T>G p.?	NC_000005.9:g.176940684A>C	Hz	Maternal	Likely Pathogenic	PVS1_VS, PM2_Sup	*DDX41*-related hematologic malignancy predisposition syndrome / AD
PKIG02013	HP:0007476 anhidrotic ectodermal dysplasia; HP:0000970 anhidrosis; HP:0000958 dry skin; HP:0002721 immunodeficiency; HP:0002715 abnormality of the immune system; HP:0005390 recurrent opportunistic infection; HP:0010701 abnormal immunoglobulin level; HP:0000498 blepharitis; HP:0002910 elevated hepatic transaminases; HP:0011496 corneal neovascularization; HP:0002091 mild restrictive lung disease; HP:0000410 mixed hearing impairment	Diagnostic Finding	*IKBKB* / HGNC:5960	NM_001556.3:c.512A>G p.(Lys171Arg)	NC_000008.10:g.42163895A>G	Hz	*De novo*	Likely Pathogenic	PS2_Strong, PS3_Sup, PM2_Sup, PP4_Sup	Immunodeficiency 15A; Immunodeficiency 15B / AD; AR
		ACMG Secondary Finding	*APOB* / HGNC:603	NM_000384.3:c.11330C>A p.(Ser3777Ter)	NC_000002.11:g.21228410G>T	Hz	Maternal	Pathogenic	PVS1_Strong, PP1_Strong, PM2_Sup, PP4	Familial hypercholesterolemia 2; Hypobetalipoproteinemia / AD; AR
PKIG02075	HP:0012181 entrapment neuropathy; HP:0012432 chronic fatigue; HP:0001369 arthritis; HP:0002650 scoliosis; HP:0002315 headache; HP:0008419 intervertebral disc degeneration	Diagnostic Finding	*LOX* / HGNC:6664	NM_002317.7:c.1035+1G>A p.?	NC_000005.9:g.121409707C>T	Hz	Unknown	Likely Pathogenic	PVS1_Strong, PM2_Sup	Familial thoracic aortic aneurysm 10 / AD
		Other Finding Unrelated to Phenotype	*SLC7A9* / HGNC:11067	NM_014270.5:c.544G>A p.(Ala182Thr)	NC_000019.9:g.33353427C>T	Hz	Unknown	Likely Pathogenic	PM6_Strong, PS3_Sup, PP4_Sup	Cystinuria / AD; AR

Abbreviations: ACMG, American College of Medical Genetics and Genomics; HGNC, HUGO Gene Nomenclature Committee; Hz, Heterozygous; Hm, Homozygous; Hp, Heteroplasmic; Hemi, Hemizygous; VS, very strong; Mod, moderate; Sup, supporting; AD, autosomal dominant; AR, autosomal recessive; XL, X-linked.
